# Investigating Factors Associated with Employees’ Attitudes Towards Work-Related Infection Control Measures During the COVID-19 Pandemic: An Exploratory Cross-Sectional Study from Seven Different Companies in Germany, July–August 2021

**DOI:** 10.3390/healthcare13192454

**Published:** 2025-09-27

**Authors:** Esther Rind, Martina Michaelis, Michael Brosi, Jana Soeder, Anna T. Neunhoeffer, Anke Wagner, Monika A. Rieger

**Affiliations:** 1 Institute of Occupational and Social Medicine and Health Services Research, University Hospital Tuebingen, University of Tuebingen, Wilhelmstraße 27, 72074 Tuebingen, Germany; martina.michaelis@med.uni-tuebingen.de (M.M.); michael.brosi@med.uni-tuebingen.de (M.B.); jana.soeder@med.uni-tuebingen.de (J.S.); anna.neunhoeffer@med.uni-tuebingen.de (A.T.N.); anke.wagner@med.uni-tuebingen.de (A.W.); monika.rieger@med.uni-tuebingen.de (M.A.R.); 2 Research Centre for Occupational and Social Medicine (FFAS), 79098 Freiburg, Germany

**Keywords:** workplaces, SARS-CoV-2 infection control measures, employee attitude, cross-sectional study, Germany, organisational and occupational health services research, workplace health management, pandemic preparedness

## Abstract

**Background/Objectives:** This study is part of an exploratory mixed-methods project investigating how companies and their employees in Germany dealt with adapted working conditions during the COVID-19 pandemic. Here, we identify predictive factors for employees’ attitudes towards the suitability of work-related technical, organisational, and personal SARS-CoV-2 infection control measures. **Methods**: In July 2021, when there was little evidence to suggest that the risk of work-related exposure to SARS-CoV-2 differed between occupations and workplaces, a standardised online and an optional paper-and-pencil survey were distributed across seven companies in southern Germany. Multivariate linear regression was used for analysis. **Results**: A total of 821 employees participated (average response rate: 24.5%). Most of the respondents (93%) worked in large companies, in the production industry (82%), with most of them having office jobs (82%). Around 29% reported doing most of their office work remotely during the pandemic. The perceived suitability of workplace infection control measures was rated quite high, with an overall mean score of 4.11 (SD 0.60) out of a possible 5. Workplace characteristics related to the COVID-19 pandemic as well as individual perception of SARS-CoV2 and COVID-19 in general were the most prominent predictors of attitudes towards the suitability of work-related SARS-CoV-2 infection control. For example, a higher COVID-19-specific reactance was negatively associated with attitudes towards technical (ß = −0.16), organisational (ß = −0.14), and personal (ß = −0.17) infection control measures (all *p*-values < 0.001). Furthermore, a higher rating of the employer’s commitment to occupational safety and health related to SARS-CoV-2, a higher individual disease perception, and a higher individual COVID-19-specific resilience had a positive association with attitudes towards the suitability of infection control measures. Finally, professional activity as well as company affiliation had statistically significant associations with employees’ attitudes towards the suitability of infection control measures. **Conclusions**: The results provide insight into factors relevant to pandemic prevention and control. In particular, our findings highlight the potential to implement organisational measures alongside compulsory technical occupational health measures. This could inform the development of pandemic preparedness strategies that prioritise adherence to established occupational infection control measures.

## 1. Introduction

On 11 March 2020, the World Health Organisation (WHO) declared the COVID-19 outbreak as a global pandemic [1]. Over the next two and a half years, hospitals, nursing homes, and other healthcare facilities made unprecedented efforts to protect patients, residents, and staff. In addition, the closure of educational institutions, public facilities, and businesses deemed non-essential was accompanied by the implementation of protective and hygienic measures in all areas of life to contain the spread of SARS-CoV-2.

Infection control measures have been designed to protect the health and safety of the public and the workforce, but they have also been perceived as restrictive and burdensome [2]. In Germany, the Occupational Health and Safety Act [3] governs the employer’s responsibility for occupational health, and the German Infection Protection Act [4] governs the national government’s responsibility for public health. During the pandemic, the SARS-CoV-2 Occupational Safety and Health Standard [5], the SARS-CoV-2 Occupational Health and Safety Regulation [6], and SARS-CoV-2 Occupational Health and Safety Ordinance [7] were the legal basis for implementing infection control measures in the workplace.

When this study’s data were collected, there was little evidence that the risk of work-related exposure to SARS-CoV-2 varied between different occupations and workplaces. The risk was particularly high for workers in healthcare, food packaging and processing, and manufacturing and office sectors [8,9]. Previous research has also shown that the workplace may be an environment with the potential to enhance or mitigate the spread of SARS-CoV-2 [8]. To minimize the spread of the virus, a number of protective measures have been recommended by governments and health and labour organisations. First, there are preventive behavioural measures: e.g., ‘wash your hands, wear a mask, keep your distance, get vaccinated’ [10]. Second, there are structural measures according to the hierarchy of industrial hazard controls [11], which include the implementation of technical and organisational protective measures and the provision of personal protective equipment.

Previous international research has shown that attitudes towards infection control interventions influence the adoption and implementation of compliant prevention behaviours [12,13]. The results of a German survey of 724 safety and health professionals conducted in 2020 reported a relatively positive picture of the acceptance of work-related infection control measures [14]. The report highlighted that the competence and motivation of employers and employees played a crucial role in the effective implementation of infection control measures. However, the frequently changing epidemiological conditions and policy adjustments required during the pandemic challenged the motivation, flexibility, and compliance of company owners, managers, and employees, sometimes referred to as “pandemic fatigue” or “natural response to a prolonged public health crisis” [4,15]. In addition, research has shown that compliance with COVID-19-related infection control measures varies among workers in different industries and is relatively high, for example, in the food and hospitality sector compared with transport and logistics occupations [16].

In early 2020, we designed a comprehensive explorative modular mixed-methods approach (please see study protocol for more details on the study design) [17] based on principles rooted in occupational and organisational health services research [18,19], to specifically address individual, organisational, and other relevant determinants of worker health during the course of the COVID-19 pandemic. At the time of the study design, there was little empirical evidence on factors that predict workers’ attitudes towards COVID-19-related infection control measures. This study is part of and builds on the results of the project “Supporting companies in the implementation and acceptance of infection control measures and changed working conditions in the context of the COVID-19 pandemic’’, which was funded by the Ministry of Science, Research, and Art, Baden-Wuerttemberg, between April 2021 and December 2022 [17,20]. In addition, we used our own institutional funding.

First, we conducted three repeated employee surveys (August–October 2020, January 2021, October–November 2021) at six German company sites of a worldwide leading global supplier of technology and services. Overall, attitudes towards infection control measures were positive and associated with sociodemographic factors (e.g., age), perceptions of the impact of the pandemic in the workplace (e.g., trust in colleagues to follow distancing rules), feeling well-informed about possible work-related infection risks, the perceived psychosocial demands through factors related to the work environment, the perceived commitment of the management to safety and health, and general attitudes towards COVID-19 (e.g., resilience) (please see Supplementary Material for other studies related to this article).

Second, we applied a mixed-methods approach combining data from two employee surveys (n = 652) and interviews with 10 key actors responsible for organising a pilot workplace vaccination programme to retrospectively assess attitudes and participation of employees and key health and management personnel regarding the implementation of the programme in five German companies in May and June 2021. The programme was generally well received, and participants highlighted the positive role of occupational health services in managing the COVID-19 pandemic, as well as the high organisational and administrative burden (please see Supplementary Material for other studies related to this article).

Third, we conducted a cross-sectional survey at six state-approved higher education institutions in Southern Germany between July and November 2021. Attitudes towards behavioural (e.g., social distancing) and structural (e.g., hybrid lecture formats) measures to prevent infections with SARS-CoV-2 were very positive, and higher education students showed a higher level of vaccine acceptance compared to the general population (please see Supplementary Material).

What is still lacking is a better understanding of common predictors of workers’ attitudes towards the suitability of technical, organisational, and personal work-related infection control measures, considering individual, workplace, and COVID-19-related factors across different companies. This is relevant not only in the context of the COVID-19 pandemic, but also for the development of strategies to promote compliance with established occupational safety and health measures in different occupations and work environments. The subsequent research question guided our study:

Which factors predict employees’ attitudes towards the suitability of recommended SARS-CoV-2 infection control measures in the summer of 2021?

## 2. Materials and Methods

### 2.1. Study Design

As we were interested in employees’ attitudes towards infection control measures, it would have been appropriate to directly observe the implementation of these measures, using, for example, an ethnographic study design, e.g., a combination of participant observation and interviews with managers and workers. However, site visits were not permitted during the pandemic. We therefore opted for a cross-sectional study design, as this would provide the best possible evidence on factors predicting attitudes towards COVID-19-related infection control measures. Due to the codex of good scientific practice and the framework for developing and evaluating complex interventions [21], we continued the exploratory approach throughout the overall study design. Despite the various challenges posed by the pandemic to managers, workers, and their families, the participating companies kindly agreed to take part in a single wave of the survey in the summer of 2021.

This study was approved by the responsible Ethics Committee of the Faculty of Medicine, University of Tuebingen, University Hospital Tuebingen in June 2020 (No.: 423/2020BO). This manuscript was reported according to the STROBE checklist for cross-sectional studies (see Supplementary Table S1) [22].

### 2.2. Epidemiological Context During Data Collection

During data collection (July–August 2021), incidence rates in Germany were relatively low, but already increasing, leading to the fourth wave of the COVID-19 pandemic (variant of concern: Delta). Regarding the political and legal context, the Occupational Health and Safety Ordinance [23] (renewed version from 25 June 2021) was in force, requiring employers to perform a COVID-19-related risk assessment, develop an occupational hygiene concept, and reduce work-related contacts. To further reduce the risk of infection in the workplace, the employer was required to provide free SARS-CoV-2 infection testing to on-site workers at least twice a week, unless other sufficient protective measures were in place (e.g., arranging digital meetings, maintaining social distancing, and wearing appropriate personal protective equipment).

### 2.3. Recruitment and Data Collection

In the early summer of 2021, we invited a total of 14 companies located in the southwest of Germany to participate in an employee survey on the topic of “occupational safety and health in the context of the COVID-19 pandemic”. In accordance with the European Commission’s recommendation on enterprise size definition [24], our aim was to include small- (staff < 50) and medium-sized (staff < 250) as well as large companies (≥250 employees). We used convenience sampling [25], drawing on our extensive corporate and occupational health network, which includes companies from a range of industries (e.g., chemical and pharmaceutical industry; production industry; logistics; see Supplementary Table S4 for more detailed information). We called all companies and asked to speak to company management. We also distributed written study invitations and shorter study information (flyer) to the company owners, managers, and employee representatives in close collaboration with the responsible occupational health service, who in turn informed the workforce. On average, we contacted each company three times. The reasons for refusal were primarily related to a lack of resources, particularly “time” and “personnel to support the study team”, as well as “economic pressure”.

Inclusion criteria for employees were a minimum age of 18 years, the ability to complete the voluntary survey in German, and written consent given at the beginning of the online survey. It was not possible to continue with the anonymous online survey without consenting to participate at the beginning of the questionnaire. Employees who wanted to participate in the anonymous paper-and-pencil survey returned the questionnaire via sealed ballot boxes set up at the participating companies, which were then collected by the study team. Data were collected in July and August 2021 using a largely standardized and pre-tested questionnaire developed using the established survey tool Unipark [26], with an optional paper-and-pencil version to encourage employees without computer/mobile access to complete the survey during working hours. The link to the online survey was forwarded to employees by their respective company representatives. Paper questionnaires could be dropped into sealed ballot boxes provided at central locations within the companies. A reminder was sent to the companies one week after the initial invitation. The survey took approximately 25 min to complete. At the end of the survey period, the paper-and-pencil questionnaires were scanned by the research study team [27] and the data merged with those from the online survey.

### 2.4. Measures: Outcome and Potentially Predictive Variables

The development of the employee survey was described in detail elsewhere [28]. In short, the questionnaire included self-developed items and questions from previous studies [5,29,30,31,32,33,34,35,36,37,38,39,40,41,42,43].

The outcome ‘attitudes towards the suitability of work-related infection control measures’ (wICM), which were implemented for infection control at the different company sites, was measured by 26 items according to the German SARS-CoV-2 Occupational Safety and Health Standard [5]. The original question was ‘‘How suitable do you consider the following recommended measures to prevent the spread and infection of the novel coronavirus in the workplace?”

Participants rated the suitability of each item along a 5-point Likert scale ranging from ‘not at all suitable’ to ‘very suitable’. A mean score according to the mean-across-available-item approach [44] was calculated for further statistical analysis (for more details, please see the section on statistical analysis). High values indicate a positive attitude towards wICM. Besides the total wICM score, subscores were constructed and grouped according to the hierarchy of control for technical (10 items), organisational (6 items), and personal measures (10 items) (see Supplementary Table S2) [5,28]. The internal consistency was good, with Cronbach’s α = 0.92 (total wICM score), and α = 0.79 (personal measures), α = 0.80 (organisational measures), and α = 0.81 (technical measures).

Included variables that were potentially predictive of the main outcome covered the subsequent subject areas as categorized in previous publications [28] (please see Supplementary Material for other studies related to this article):

(I) sociodemographic characteristics (e.g., age, gender, education), also including Big Five personality traits [34] and social desirability [41];
(II) general workplace characteristics: company characteristics (e.g., company size, industry sector) and job-related characteristics (e.g., leadership position, professional activity);
(III) workplace characteristics related to the COVID-19 pandemic (e.g., perceived psychosocial demands during the pandemic, working full-time);
(IV) perception of SARS-CoV-2 and COVID-19 in general (e.g., disease perception, COVID-19-specific resilience);
(V) variables relating to SARS-CoV-2 rapid antigen testing and COVID-19 vaccination (e.g., potential readiness to perform SARS-CoV-2 rapid antigen tests, perceived benefit of COVID-19 vaccination).

### 2.5. Statistical Analysis

Descriptive results of the raw data were reported as percentages and means (M) ± standard deviations (SDs). For further multivariate analysis, missing values of less than 6% were imputed using IBM SPSS version 28. Due to a higher number of missing values, this procedure could not be applied to the questions about age, previous SARS-CoV-2 infections, and the subjectively perceived change in sick leave during the COVID-19 pandemic. These variables were therefore excluded from further statistical analysis.

Mean scores were calculated for previously validated scales according to their manuals. Because the original study design was exploratory rather than hypothesis-driven [17], we included plausible predictors of attitudes towards work-related SARS-CoV-2 infection control measures based on our previous research [28] (please see Supplementary Material for other studies related to this article). To keep the number of variables manageable, mean scores were also calculated for different items covering variations of the same aspect. This included the following (see Supplementary Table S3):

- trust in colleagues’ adherence to distance and hygiene rules (2 items, Cronbach’s α = 0.93);
- perceived personal susceptibility and expected severity of infection with SARS-CoV-2 in private surroundings (3 items, Cronbach’s α = 0.63);
- perceived benefit of COVID-19 vaccination for the individual or the company (3 items, Cronbach’s α = 0.64).

Variables were coded as metric variables, categorical variables with a defined reference category, or dummy coded variables with 0 = no and 1 = yes.

Mean differences between the three subscales were calculated using the nonparametric Wilcoxon rank-sum test, and the associated effect size r was calculated from the standardized test statistic z divided by the square root of the number of cases. Values < 0.3, ≥ 0.3 and ≤ 0.5, and > 0.5 were considered small, moderate, and strong effects, respectively [45].

We subsequently analysed overall differences between companies regarding ‘attitudes towards the suitability of work-related SARS-CoV-2 infection control measures’ (wICM and subscores) using bivariate linear regression. To assess the statistical effects of potential predictor variables on the outcomes, we conducted a two-step linear regression analysis. First, we performed an exploratory bivariate regression analysis for the total wICM score and the three subscores. We then considered the independent variables with *p*-values < 0.02 for a multivariate regression analysis. Next, we used the backward method to eliminate variables (*p*(out) = 0.051) in order to achieve a parsimonious model. We categorised a *p*-value of ≤ 0.05 (two-sided) as statistically significant, classifying the standardised regression coefficient beta as indicating a small, medium, or large effect (0.10–0.29, 0.30–0.49, and ≥0.50, respectively) [46,47]. We adjusted our multivariate analysis for professional activity (assembly line/production/warehouse; office on-site; office remote) and individual company (n = 7).

## 3. Results

### 3.1. Characteristics of the Sample

In accordance with the European Commission’s recommendation on the definition of micro, small, and medium-sized enterprises [24], two medium-sized (50 to <250 employees) and four large companies (≥250 employees) participated in this study (N = 3632 according to information provided by each of the participating companies). The average response rate was 24.5% (SD 10.7%; range 12.0%–42.6%); 821 respondents were included in the analysis. More detailed characteristics of all seven companies (response rate by industry sector and type of affiliated statutory accident insurance) can be found in Supplementary Table S4.

The sociodemographic characteristics of the employees surveyed are described in Table 1, for example, most employees were aged 30–49 (46.4%), male (56.0%), born in Germany (90.6%), and had a higher education (69.1%). The majority of the respondents (93.2%) worked in large companies, in the production industry (81.7%), had office jobs (82.3%), and 23.5% were in managerial positions. Most of the participants worked full-time (84.0%) and on-site (17.7%: assembly line/production/warehouse and 53.7%: office jobs). A further 28.6% of the respondents reported doing most of their office work from home during the pandemic. A summary of all potential predictors of the main outcome, ‘attitudes towards the suitability of work-related SARS-CoV-2 infection control measures’, is presented in Supplementary Table S3.

### 3.2. Attitudes Towards the Suitability of Work-Related SARS-CoV-2 Infection Control Measures

On a scale of 1 to 5, the participants rated the suitability of the recommended work-related SARS-CoV-2 infection control measures as relatively high (total wICM score: mean = 4.11, standard deviation [SD] = 0.60). The lowest and highest values were 3.89 (SD 0.68) and 4.51 (SD 0.38) in companies 7 and 3, respectively (see Table 2). Regarding the differences between the subscores, personal measures received the highest approval (mean = 4.26, SD = 0.57), followed by technical measures (mean = 4.04, SD = 0.68) and organisational measures (mean = 3.97, SD = 0.80). The effect sizes for the differences between the personal and technical subscores (r = 0.40, *p* < 0.001) and the personal and organisational subscores (r = 0.44, *p* < 0.001) were medium. The difference between the technical and organisational measures was small (r = 0.13, *p* < 0.001). Compared to employees in companies 1, 2, and 7, employees in companies 3, 4, and 5 had significantly more positive attitudes towards the suitability of infection control measures (wICM total score, technical and organisational subscores). Employees of company 6 also had a more positive attitude towards the suitability of organisational infection control measures. The results were also statistically significant regarding attitudes towards personal infection control measures for employees in companies 3 and 5.

### 3.3. Factors Associated with Attitudes Towards Work-Related SARS-CoV-2 Infection Control Measures

The results of the bivariate analysis are presented in Supplementary Table S5. Only variables with *p*-values < 0.02 were included in the multivariate analysis. Table 3 shows all the variables that were found to significantly predict “attitudes towards the suitability of work-related infection control measures”. The linear regression with multiple explanatory variables explained 37% of the total variance in the dependent variable “wICM” (total score), and 32%, 30%, and 36% of the total variance in the technical, organisational, and personal subscores, respectively. The effect sizes for all statistically significant predictors were small (beta < 0.25).

The highest effect sizes across the total score (wICM) and all three subscores were two variables from the category “workplace characteristics related to the COVID-19 pandemic”. The higher the reported COVID-19-specific reactance, the more negative the rating of the suitability of work-related infection control measures (wICM: beta = −0.18, *p* < 0.001). However, the higher the employees’ rating of the employers’ SARS-CoV-related occupational safety and health commitment, the more positive the rating of the suitability of the work-related infection control measures (wICM: beta = 0.17, *p* < 0.001). Furthermore, a higher disease perception was associated with a more positive attitude towards the infection control measures. A high COVID-19-specific resilience was also associated with a more positive attitude towards infection control measures, and the effect size was relatively high (beta = 0.20, *p* < 0.001) for organisational infection control measures compared to the total score (wICM: beta = 0.12, *p* < 0.001) and the technical and personal subscores (beta = 0.09, *p* = 0.005 and beta = 0.12, *p* < 0.001, respectively).

Regarding employees’ professional activities, the results show that workers on the assembly line, in production, or in the warehouse, as well as office personnel working on-site, had more negative attitudes towards the suitability of work-related infection control measures than office personnel working remotely. The effect size was strongest for the association between workers on the assembly line, in production, or in the warehouse, and work-related organisational (organisational subscore: beta = −0.24, *p* < 0.001) and technical infection control measures (technical subscore: beta = −0.18, *p* < 0.001). Due to the varying response rates among the seven companies and for reasons of confidentiality, we cannot present differences between the companies in more detail. However, we can report that the results suggest a significant ‘company effect’ in relation to the associations between three companies and both the total wICM score and the organisational subscore. One company showed significant associations with the total wICM score and all subscores; however, the number of cases in this group was relatively low compared to the other companies. Therefore, the estimate is considered to be less stable, which is also indicated by a wide confidence interval.

Variables excluded from the multivariate regression models due to *p*-values > 0.02 in the bivariate analysis (see Table S5) or *p*-values > 0.05 in the multivariate analyses:

I. Sociodemographic characteristics: country of birth; living in a committed relationship; household size; children younger than 18 years living in the household; health professional living in the household; personality traits “extraversion”, “openness” and “conscientiousness”; social desirability of exaggerating positive qualities;
II. General workplace characteristics: company size; industry sector; affiliated statutory accident insurance; employment at company (years); shift work; short-time work; fixed-term contract;
III. Workplace characteristics related to the COVID-19 pandemic: perceived psychosocial demands from aspects of work content, work organisation and work environment; perceived probability of contracting COVID-19 in the workplace;
IV. Perception of SARS-CoV-2 and COVID-19 in general: perceived adequacy of media coverage of SARS-CoV-2; frequency of self-information about SARS-CoV-2; perceived personal susceptibility and expected severity of infection with SARS-CoV-2 in private surroundings; affiliation to risk group for developing severe COVID-19 disease; frequent contact with individuals from the risk group; (inter)national travelling and recreational activities with an increased risk of infection; knowledge about confirmed cases of infected peers;
V. Variables relating to SARS-CoV-2 rapid antigen testing and COVID-19 vaccination: antibody test ever performed; positive rapid antigen test result obtained; potential readiness for COVID-19 vaccination within the next week.

## 4. Discussion

At the very beginning of the COVID-19 pandemic, when no studies were yet available to draw upon, we pursued an interdisciplinary theory-based deduction of plausible subject areas and variables to predict employees’ attitudes towards the newly implemented work-related infection control measures [17]. Hence, we did not predefine a specific working hypothesis but chose an exploratory approach. Based on our previous research [28] (please see Supplementary Material for other studies related to this article), we considered a variety of individual (e.g., sociodemographic and socio-economic variables, individual risk perception, resilience, attitudes towards antigen testing and vaccination) and organisational factors (company as well as job- and workplace-related characteristics regarding the COVID-19 pandemic) to draw a comprehensive picture on how companies implement and employees perceive working conditions in Germany during the COVID-19 pandemic. This is relevant not only in the context of the COVID-19 pandemic, but it may also inform future studies aiming to facilitate and enhance compliance with evidence-based occupational health and safety measures. This, in turn, improves the management of new and ongoing infectious diseases (e.g., different types of influenza), which is crucial for public health and economic stability.

### 4.1. Comparison to Other Studies

In this study, we particularly focussed on factors predicting employees’ attitudes towards the suitability of recommended SARS-CoV-2 infection control measures in the summer of 2021 across different companies. We assessed attitudes regarding the suitability of work-related COVID-19 infection control measures according to the hierarchy of industrial hazard controls [11], which include the implementation of technical and organisational protective measures and the utilisation of personal protective equipment. In line with a previous cross-sectional [28] and a repeated cross-sectional analysis (please see Supplementary Material for other studies related to this article), based on data from employees across six German company sites of a worldwide leading global supplier of technology and services, employees of the participating companies in this study rated the suitability of work-related COVID-19 infection control measures as relatively high, with the highest rating of personal measures, followed by technical and organisational measures. In comparison to office personnel working remotely, we also observed a more negative attitude towards work-related COVID-19 infection control measures for office personnel working on-site and particularly for workers on the assembly line, in production, or in the warehouse regarding organisational infection control measures.

Regarding the hierarchy of industrial hazard control, which emphasises a higher effectiveness of technical compared to organisational and personal measures [49], our findings highlight the potential of organisations to intensify the implementation and promotion of organisational alongside mostly compulsory technical occupational health measures as an important part of a comprehensive workplace health management. The importance of organisational support (and knowledge about COVID-19) regarding COVID-19 preventive behaviour has, for example, also been demonstrated among Thai construction workers [50]. In comparison, previous research from Germany emphasised the stronger implementation of personal measures compared to organisational measures [14,16,51] as well as differences in attitudes towards infection control measures across different occupations [16]. Although the majority of respondents of another German employee survey (survey period: January–February 2021) rated the implemented work-related infection control measures as “adequate” (relative frequency: 85%), there were differences between occupational groups, with the highest rating from employees in occupations in commerce and trade as well as IT and natural science services occupations (relative frequency: 93%) and the lowest rating from employees in social-cultural service occupations (77%). The number of employees in manufacturing occupations rating work-related infection control measures as “adequate” was also relatively high (90%); however, there was no further information on the professional activity of the respondents (e.g., office job or working at the assembly line) [16].

Regarding factors associated with attitudes towards work-related COVID-19 infection control measures, all detected significant predictive variables had at least small effects. In terms of workplace characteristics related to the COVID-19 pandemic, a higher trust in colleagues’ adherence to distance and hand hygiene rules as well as a higher rating of the employer’s commitment to SARS-CoV-2-related occupational safety and health measures was associated with a more positive attitude towards the suitability of work-related personal infection control measures. Previous studies have demonstrated the importance of trust in the implementation and acceptance of occupational safety measures, which is rooted in the commitment of employers and employees alike to effectively manage and adhere to existing safety measures [52,53]. Furthermore, our results show that a lower COVID-19-specific reactance was associated with a more positive attitude toward the suitability of work-related infection control measures across the total wICM score and all three subscores. Interestingly, the COVID-19-specific reaction in our sample was slightly lower (mean: 3.2) compared to the general population in Germany in July/August 2021 (mean: 3.7) [54]. This may be related to a higher trust in work-related infection control measures compared to the prevailing trust in public institutions. For example, in July/August 2021, the general populations’ trust in the German Government and the media was lower compared to their trust in health and academic institutions [54]. Our results, however, indicate a lower reactance and a relatively high trust in workplace characteristics, including colleagues’ adherence and the employers’ commitment to infection control measures. As reactance has been shown to influence preventive behaviour, governments could promote the development of public health messages, and in the workplace, the implementation of work-related occupational safety and health measures that mitigate reactance and encourage compliance with safety measures. This, in turn, could strengthen the overall resilience of individuals and organisations [55].

Finally, we observed differences in employees’ attitudes towards work-related COVID-19 infection control measures across some of the companies, primarily with regard to the total wICM score and the organisational subscore. This is important because, unlike technical measures, which are highly regulated and therefore generally apply to all companies, organisational measures provide business owners and executive managers with more flexibility to build a positive occupational health climate and culture [56,57], prioritising the development and management of targeted organisational preventive measures for their specific working environment. Only one company had significant associations with the total wICM score and all subscores which may be related to contextual factors regarding the relatively small company size (<100 employees), a high proportion of the workforce working in production compared to relatively few office personnel, and a family-run management potentially pursuing a relatively comprehensive workplace health management strategy.

### 4.2. Strength and Limitations

A key strength of this study is its transdisciplinary exploratory approach, which was carried out in the early stages of the pandemic, including seven companies of different sizes and from different industry sectors facilitating the transferability of our findings to similar industrial contexts. The recruitment of these companies during the beginning of the pandemic with constantly changing guidelines, regulations, lockdowns, and travel restrictions was time-consuming and challenging. As we were not permitted to visit the participating companies in person to observe how infection control measures were implemented and managed, we partly adapted the original questionnaire developed with employees of a global supplier of technology and services in Germany [28], alongside our on-site contact persons, to capture the working environment at the seven companies as accurately as possible. Once the data had been collected, the companies received short reports. These reports provided company management with information they needed to take practical measures to optimise occupational health and safety for their employees.

Nevertheless, this study has several limitations [28] (please see Supplementary Material for other studies related to this article). As we relied on convenience sampling [25], the participation of respondents with a predominantly positive attitude towards infection control measures (selection bias), as well as the elicitation of self-report measures (response bias), must be considered. Due to the online format of our survey, which was available only in German, it is likely that we excluded a number of employees who might otherwise have participated. This probably explains why more office staff than production line or warehouse workers participated in this study. Future studies may consider the need for oversampling blue-collar workers. The latter group was particularly affected by the implementation of infection control measures due to the nature of their professional activity, which required working on-site, frequently with close contact to co-workers. Recently, a number of studies have demonstrated an increased risk of infection for a variety of occupational professions. This includes, for example, workers in professions declared as ‘essential’, such as healthcare and medical professionals, cleaning services workers, food sellers, meat industry workers, public transport workers, warehouse and logistics workers, and teachers, where personal contact with patients, students, and customers is required [58,59,60]. A recent systematic review by Gabriel et al. [61] focusses on the SARS-CoV-2 infection risk by non-healthcare occupations and identifies a statistically increased risk of being infected for occupations in five sectors: food and hospitality, social services, transport and logistics, security and surveillance, and cleaning. Furthermore, studies have demonstrated an increased risk of infection for seasonal migrant workers [62,63,64]. While these studies all contribute to a better understanding of occupational differences in the risk of infection, none of them provide evidence regarding attitudes towards the suitability of infection control measures, which has been shown to impact compliant prevention behaviours [12,13]. Furthermore, this study’s questionnaire was specifically developed and has not yet been validated.

Unfortunately, we were unable to recruit small enterprises, which, compared to medium and large enterprises, may have fewer resources available for the implementation and management of infection control measures. Results from a mixed-methods study investigating the impact of the COVID-19 pandemic on small businesses and workers suggest that smaller businesses, including beauty salons and auto repair shops in Southern Arizona, were, among other things, affected by conflicts over suitable health and safety protocols or a lack of clear guidance from policymakers. These issues are also transferable to larger companies as well as to the German context. This study concludes that sustained educational outreach and financial support, both factors that can be addressed at the policy and institutional level as well as at the organisational level, are likely to strengthen the implementation of healthy and safe work practices [65]. Additionally, as our study was conducted in Germany during a specific phase of the pandemic, the results may not be fully applicable to other countries or later stages of the pandemic, during which time attitudes and organisational practices may have evolved differently. Finally, due to the cross-sectional nature of the data, it is not possible to draw causal inferences.

### 4.3. Meaning of the Study, Implications, and Future Work

During the COVID-19 pandemic, the public and political focus regarding pandemic management was primarily on healthcare professionals, who were, of course, under a great deal of stress and strain [66]. At the time, the issue of attitudes towards the suitability of infection control measures in different companies with production was not widely discussed in public, particularly in the early stages of the pandemic. However, our focus on production remains relevant, particularly in terms of preparing for future pandemics and dealing with ongoing health challenges effectively, such as the regular spread of influenza viruses.

The lessons learnt from the COVID-19 pandemic have emphasised the importance of developing and enhancing pandemic preparedness beyond this pandemic at the macro (institutional), meso (organisational), and micro (individual) level. A key aspect of this is promoting the acceptance of general occupational safety measures. There is increasing evidence that participatory approaches [67], which include target populations in the study process, provide relevant and viable suggestions for developing tailored interventions for specific working environments [68]. A positive implementation climate that incorporates salutogenetic principles, such as relevance, meaning, confidence, ownership, and trust, facilitates the development, implementation, and management of occupational health measures. It also increases compliance among those affected [69]. Furthermore, this approach promotes and includes the consideration of public health ethics principles (e.g., treating people with concern and respect, minimising harm and working together) [70], as well as social determinants of infection risk, susceptibility, and disease severity (e.g., living and working conditions, organisational and individual health literacy), when developing interventions that contribute to enhanced pandemic preparedness [71].

With respect to the broader context of occupational health and safety, a study by Jain et al. (2022) [72] raises the question of whether existing organisational measures are frequently geared more towards promoting individual resources than towards improving work organisation and workplace design. Although their study focusses on dealing with work-related stress, the implications are similar for the context of this study. Firstly, focussing primarily on strengthening individual resources in the face of increasing work demands, psychosocial stress [73], as well es external stressors (e.g., the COVID-19 pandemic) is problematic because it provides individual solutions to systemic and structural problems. The Total Worker Health^®^ (TWH) framework, for example, provides a holistic approach that focusses on the impact of work on health and general well-being, taking into account physical, psychological, social, and economic aspects [74].

Secondly, we argue that inadequate occupational health management, including the insufficient implementation of infection control measures, could be considered a psychosocial stressor for employees, managers, and business owners, all of whom are responsible for the implementation of workplace health measures. A recent realist review focussing on participatory workplace intervention to improve work-related musculoskeletal health emphasised the importance of creating a meaningful and trusting intervention climate involving individuals at all organisational levels, which requires a clear distribution of responsibilities as well as the allocation of sufficient monetary and human resources [69].

Although there is increasing evidence of the effectiveness of complex interventions that combine organisational [75] and behavioural preventive elements, the current evidence remains relatively limited, inconsistent, and poorly systematised [76]. Future research may increasingly adopt longitudinal and mixed-method designs. For example, these could include stratified random sampling, the targeted recruitment of smaller enterprises, or the investigation of attitudes towards the suitability of infection control measures in different post-pandemic preparedness settings, in order to track how attitudes evolve in response to changing working conditions and existing workplace health management strategies. As the effect sizes of the plausible predictors included in this study were rather modest, larger and more diverse samples could improve the representativeness and the transferability of the results. Furthermore, qualitative studies could explore the reasons behind observed attitudinal differences among occupational groups and different companies in more depth, thereby informing targeted preventive strategies. We recently published a longitudinal mixed-methods study from Germany (please see Supplementary Material for other studies related to this article) exploring how a company group dealt with challenges related to the COVID-19 pandemic and how employees and managers perceived work-related psychosocial demands. The results highlight the importance of pandemic preparedness, particularly the qualitative findings, which emphasise the need for a crisis management team, a culture of trust, transparent communication, and participatory approaches to change processes. Extending the research to different industries and cultural contexts would enrich our understanding and help us strengthen workplace health management overall.

## 5. Conclusions

In our data, the factors that best predicted employees’ attitudes towards the suitability of recommended SARS-CoV-2 infection control measures in the summer of 2021 were general workplace characteristics (professional activity), COVID-19-related workplace characteristics (trust in colleagues’ adherence to distance and hygiene rules, COVID-19-specific reactance, employees’ rating of employers occupational safety and health commitment), and perception of SARS-CoV-2 and COVID-19 in general (disease perception and COVID-19-specific resilience). Regarding the workplace-related characteristics, our findings highlight the potential for implementing organisational measures alongside compulsory technical occupational health measures. Before demanding the implementation of personal protective measures, employers and occupational health policymakers should prioritise the promotion/funding and effective implementation of technical and organisational-level interventions. A participatory and salutogenetic approach involving transparent institutional and organisational communication, and the expertise of those affected by the implementation process, is likely to encourage acceptance of, and compliance with, occupational health measures. This is particularly important when developing and implementing occupational safety and health measures adapted to specific working environments. In turn, this is likely to strengthen institutional, organisational, and individual resilience as an important part of effective workplace health management and preparing for future global health challenges.

## Figures and Tables

**Table 1:** Sociodemographic characteristics of the sample [N = 821].

Variable [Reference]	Mean (Standard Deviation) or Percentages [n_valid_]	
Age (years) [32,40]		**[679]**
18–29	16.1%	[109]
30–49	46.4%	[315]
> 49	37.6%	[255]
*Missing values*	17.3%	[142]
Gender [32,40]		**[791]**
male	56.0%	[443]
female [Ref ^+^]	43.2%	[342]
diverse *	0.8%	[6]
*Missing values*	3.7%	[30]
Country of birth [48] ^†^		**[789]**
Germany	90.6%	[715]
other country (Ref)	8.6%	[68]
do not know *	0.8%	[6]
*Missing values*	3.9%	[32]
Education [33]		**[804]**
primary	6.1%	[49]
intermediate	24.8%	[199]
higher (Ref)	69.1%	[556]
*Missing values*	2.1%	[17]
Living in a committed relationship [32,40] ^†^		**[771]**
no (Ref)	17.8%	[137]
yes	82.2%	[634]
*Missing values*	6.1%	[50]
Household size [32,40] ^†^		**[794]**
alone (Ref)	14.2%	[113]
at least 2 persons	85.7%	[681]
*Missing values*	3.3%	[27]
Children < 18 years living in the household		**[784]**
no (Ref)	35.2%	[508]
yes	64.8%	[276]
*Missing values*	4.5%	[37]
Health professional in the household [32,40] ^†^		**[790]**
no (Ref)	89.1%	[704]
yes	10.9%	[86]
*Missing values*	3.8%	[31]

Note: Translated to English by the authors for the purpose of publication. The original version was in German only. See Soeder et al. 2022 [28]: Supplementary File S2. ^+^ Reference group. ^†^ Adapted to the target group. * Category was excluded from further analysis because n < 10.

**Table 2:** Company differences regarding ‘attitudes towards the suitability of work-related SARS-CoV-2 infection control measures’ total score (wICM) and subscores for technical, organisational, and personal measures: results of the bivariate linear regression models.

	wICM ^1^ (Total Score)		Technical Measures (Subscore)		Organisational Measures (Subscore)		Personal Measures (Subscore)	
Company 1–7 [n]	Mean (SD ^2^)	Beta (*p*) ^3^	Mean (SD)	Beta (*p*) ^3^	Mean (SD)	Beta (*p*) ^3^	Mean (SD)	Beta (*p*) ^3^
1 [41]	3.98 (0.80)	0.03 (0.460)	3.94 (0.79)	0.05 (0.295)	3.83 (0.96)	0.06 (0.202)	4.12 (0.82)	−0.01 (0.780)
2 [46]	4.06 (0.61)	0.06 (0.175)	3.89 (0.74)	0.03 (0.467)	3.86 (0.83)	0.07 (0.136)	4.33 (0.59)	0.07 (0.117)
3 [10]	4.51 (0.38)	**0.11** **(0.003)**	4.37 (0.44)	**0.09** **(0.013)**	4.50 (0.48)	**0.12** **(0.001)**	4.66 (0.34)	**0.10** **(0.010**)
4 [467]	4.10 (0.59)	**0.17** **(0.014)**	4.05 (0.67)	**0.19** **(0.007)**	3.97 (0.80)	**0.22** **(0.002)**	4.23 (0.55)	0.07 (0.339)
5 [148]	4.24 (0.55)	**0.22** **(0.000)**	4.17 (0.64)	**0.21** **(0.001)**	4.15 (0.73)	**0.25** **(0.000)**	4.38 (0.50)	**0.15** **(0.014)**
6 [54]	4.10 (0.57)	0.09 (0.051)	3.99 (0.66)	0.08 (0.097)	4.02 (0.73)	**0.12** **(0.009)**	4.26 (0.57)	0.06 (0.241)
7 [55] ^4^	3.89 (0.68)		3.79 (0.74)		3.62 (0.83)		4.16 (0.65)	
Overall	4.11 (0.60)		4.04 (0.68)		3.97 (0.80)		4.26 (0.57)	
[N]	[818]		[818]		[817]		[818]	
**Model statistics**	**R ^2^**		**R ^2^ _(corrected)_**		**F-statistics**		***p***	
wICM (total score)	0.02		0.02		3.5 (6;816)		0.002	
Subscores:								
Technical measures	0.02		0.01		3.0 (6;816)		0.006	
Organisational measures	0.03		0.02		4.2 (6;816)		<0.001	
Personal measures	0.02		0.01		2.9 (6;816)		0.009	

^1^ wICM = work-related SARS-CoV-2 infection control measures (total score). ^2^ SD = standard deviation. ^3^ Beta (*p*) = statistically significant (*p* < 0.05) [method: enter; raw data). ^4^ Reference category in the regression model.

**Table 3:** Results of the multivariate regression analysis (imputed data; n = 821) ^1^ predicting ‘attitudes towards the suitability of work-related infection control measures’.

	β (*p*) of Total Score (wICM) and Subscores (T–O–P) ^2^			
Variable	wICM ^3^ (Total Score)	Technical Measures	Organisational Measures	Personal Measures
**I. Sociodemographic characteristics (including Big Five personality traits and social desirability)**				
Gender (Ref ^4^: 0 = female and 1 = male)	– ^5^	–	–	−0.09 (0.010)
Primary education (Ref: higher)	–	–	–	0.08 (0.013)
Personality trait neuroticism (1 = low to 5 = high)	0.08 (0.006)	–	0.08 (0.008)	0.07 (0.023)
Personality trait agreeableness (ditto)				−0.07 (0.023)
Social desirability of exaggerating negative qualities (1 = not at all to 5 = very much)				0.08 (0.032)
**II. General workplace characteristics (company characteristics and job-related characteristics)**				
Work in changing teams (0 = no and 1 = yes)	–	0.06 (0.038)	–	–
Full-time job (ditto)	−0.08 (0.012)	−0.10 (0.001)	–	–
Leadership position (ditto)	0.06 (0.038)	–	–	0.07 (0.027)
Professional activity (Ref: office remote work) ^1^				
assembly line/production/warehouse (0 = no and 1 = yes)	−0.12 (0.001)	−0.18 (<0.001)	-0.24 (<0.001)	0.00 (0.952)
office on-site (ditto)	−0.06 0(0.097)	−0.08 (0.027)	−0.08 (0.026)	−0.03 (0.451)
**III. Workplace characteristics related to the COVID-19 pandemic**				
Perceived psychosocial demands from aspects of social relations during the pandemic (1 = negative to 5 = positive rating)	–	-0.11 (0.007)	–	–
Trust in colleagues’ adherence to distance and hygiene rules (1 = not at all to 7 = always)	0.13 (<0.001)	0.11 (0.001)	0.07 (0.027)	0.15 (<0.001)
Perceived self-efficacy to avoid a SARS-CoV-2 infection in the current workplace situation (1 = extremely difficult to 7 = extremely easy)	–	0.07 (0.036)	–	–
Feeling informed about possible risks of SARS-CoV-2infection at work (0 = poor/satisfactory and 1 = good/very good)	0.07 (0.036)	0.06 (0.060)	–	0.08 (0.014)
COVID-19-specific reactance (1 = not at all to 7 = very much)	−0.18 (<0.001)	-0.16 (<0.001)	−0.14 (<0.001)	−0.17 (<0.001)
Leadership quality as rated by employees (1 = lowest to 100 = highest rating)	0.07 (0.033)	0.11 (0.008)	–	–
Employees’ rating of the employer’s commitment to occupational safety and health related to SARS-CoV-2 (1 = very low to 4 = very high)	0.17 (<0.001)	0.16 (<0.001)	0.14 (<0.001)	0.16 (<0.001)
**IV. Perception of SARS-CoV-2 and COVID-19 in general**				
Disease perception (1 = low to 7 = high)	0.17 (<0.001)	0.14 (<0.001)	0.14 (<0.001)	0.18 (<0.001)
Affective risk perception (ditto)	–	–	0.07 (0.058)	–
COVID-19- specific resilience (ditto)	0.12 (<0.001)	0.09 (0.005)	0.20 (<0.001)	0.12 (<0.001)
**V. Variables relating to SARS-CoV-2 rapid antigen testing and COVID-19 vaccination**				
Potential readiness to perform SARS-CoV-2 rapid antigen tests (1 = low to 7 = high)	0.11 (0.002)	0.10 (0.005)	–	0.12 (0.001)
Use of the rapid antigen test provided by the company (0 = no and 1 = yes)	–	–	–	0.07 (0.036)
Perceived benefit of COVID-19 vaccination for the individual or the company (1 = low 7 = high)	0.08 (0.007)	0.06 (0.043)	0.09 (0.004)	0.13 (<0.001)
One or two vaccination doses received (0 = no and 1 = yes)	–	–	–	−0.08 (0.026)
**Model statistics**	**R ^2^**	**R ^2^_(corrected)_**	**F-statistics**	***p***
wICM (total score)	0.37	0.35	21.9 (20;775)	<0.001
*Subscores:*				
Technical measures	0.32	0.31	18.0 (21;808)	<0.001
Organisational measures	0.30	0.28	20.1 (16;771)	<0.001
Personal measures	0.36	0.34	18.5 (24;810)	<0.001

^1^ All models adjusted for individual companies (n = 7) and professional activity (assembly line/production/warehouse; office on-site; office remote). ^2^ β = standardized regression coefficient beta and *p* = significance value. ^3^ wICM = work-related SARS-CoV-2 infection control measures (total score). ^4^ Ref = reference category. ^5^ ‘–’ no statistically significant effect (*p* > 0.05).

## Data Availability

Due to confidentiality reasons, the dataset presented in this article is not publicly available. Requests for access to the data should be directed to Esther Rind and Monika A. Rieger (arbeitsmedizin@med.uni-tuebingen.de).

## Supplementary Materials

The following supporting information can be downloaded at: https://www.mdpi.com/article/10.3390/healthcare13192454/s1, Table S1: STROBE checklist for cross-sectional studies; Table S2: Components of the employee survey; Table S3: Potential predictors of the main outcome; Table S4: Response rate of the participating companies; Table S5: Results of the bivariate linear regression analysis. Refs. [5,22,24,28,29,32,33,34,36,38,40,41,43,48,77,78,79,80,81,82,83,84,85] are cited in the Supplementary Materials.

## Abbreviations

The following abbreviations are used in this manuscript:

wICM	work-related SARS-CoV2 infection control measures (total score)
T	work-related technical SARS-CoV2 infection control measures (subscore)
O	work-related organisational SARS-CoV2 infection control measures (subscore)
P	work-related personal SARS-CoV2 infection control measures (subscore)
Ref	Reference category
WHO	World Health Organisation

## References

[1] World Health Organization (WHO) WHO Director-General’s Opening Remarks at the Media Briefing on COVID-19—11 March 2020. https://www.who.int/director-general/speeches/detail/who-director-general-s-opening-remarks-at-the-media-briefing-on-covid-19---11-march-2020.

[2] Hajek K.V., Häfner M. (2021). Paradoxes of Reactance During the COVID-19 Pandemic: A Social-psychological Perspective. Javn. Public.

[3] Federal Ministry of Justice Act on the Implementation of Measures of Occupational Safety and Health to Encourage Improvements in the Safety and Health Protection of Workers at Work. The translation includes the amendment(s) to the Act by Article 2 of the Act of 31 May 2023 (Federal Law Gazette I No 140) [Arbeitsschutzgesetz, ArbSchG vom 31. Mai 2023 (BGBl. I Nr. 140)]. https://www.gesetze-im-internet.de/englisch_arbschg/index.html.

[4] Federal Ministry of Justice [German Infection Protection Act—20.07.2000]. Gesetz zur Verhütung und Bekämpfung von Infektionskrankheiten beim Menschen vom 20. Juli 2000 (BGBl. I S. 1045). Zuletzt geändert durch Art. 8v G v. 12.12.2023 I Nr. 359. https://www.gesetze-im-internet.de/ifsg/.

[5] Federal Ministry of Labor and Social Affairs (BMAS) SARS-CoV-2 Occupational Safety and Health Standard (SARS-CoV-2-Arbeitsschutzstandard)—16 April 2020. https://www.bmas.de/SharedDocs/Downloads/DE/Arbeitsschutz/sars-cov-2-arbeitsschutzstandard-en.html.

[6] Federal Ministry of Labor and Social Affairs (BMAS) SARS-CoV-2 Occupational Health and Safety Regulation—10 August 2020. https://www.bmas.de/DE/Service/Presse/Meldungen/2020/neue-sars-cov-2-arbeitsschutzregel.html.

[7] Federal Ministry of Labor and Social Affairs (BMAS) [SARS-CoV-2 Occupational Health and Safety Ordinance, 21 January 2021]. SARS-CoV-2-Arbeitsschutzverordnung (Corona-ArbSchV) vom 21. Januar 2021. https://www.bundesanzeiger.de/pub/publication/5QH1uegEXs2GTWXKeln/content/5QH1uegEXs2GTWXKeln/BAnz%20AT%2022.01.2021%20V1.pdf?inline.

[8] European Centre for Disease Prevention and Control (ECDC) COVID-19 Clusters and Outbreaks in Occupational Settings in the EU/EEA and the UK—11 August 2020. https://www.ecdc.europa.eu/sites/default/files/documents/COVID-19-in-occupational-settings.pdf.

[9] World Health Organization (WHO) Considerations for Public Health and Social Measures in the Workplace in the Context of COVID-19, 10 May 2020. https://www.who.int/publications/i/item/WHO-2019-nCoV-Adjusting_PH_measures-Workplaces-2020.1.

[10] World Health Organization (WHO) Advice for the Public: Coronavirus Disease (COVID-19)—Updated 18 March 2023. https://www.who.int/emergencies/diseases/novel-coronavirus-2019/advice-for-public.

[11] The National Institute for Occupational Safety and Health Hierarchy of Controls. Udated 10 April 2024. https://www.cdc.gov/niosh/hierarchy-of-controls/about/?CDC_AAref_Val=https://www.cdc.gov/niosh/topics/hierarchy/.

[12] Cheng H.C., Chang Y.J., Liao S.R., Siewchaisakul P., Chen S.L. (2021). The impact of COVID-19 on knowledge, attitude, and infection control behaviors among dentists. BMC Oral Health.

[13] Kleitman S., Fullerton D.J., Zhang L.M., Blanchard M.D., Lee J., Stankov L., Thompson V. (2021). To comply or not comply? A latent profile analysis of behaviours and attitudes during the COVID-19 pandemic. PLoS ONE.

[14] Adolph L., Eickholt C., Tausch A., Trimpop R. (2021). [SARS-CoV-2 Occupational and Infection Control Measures in German Companies: Results of a Survey of Occupational Health and Safety Experts]. SARS-CoV-2-Arbeits- und Infektionsschutzmaßnahmen in Deutschen Betrieben: Ergebnisse Einer Befragung von Arbeitsschutz-Expertinnen und -Experten.

[15] World Health Organization (WHO) (2020). Pandemic Fatigue—Reinvigorating the Public to Prevent COVID-19: Policy Framework for Supporting Pandemic Prevention and Management.

[16] Meyer S.-C., Robelski S., Tisch A., Sommer S., Schröder C. (2021). [Well Protected at Work? Occupational Safety in the Corona Pandemic from the Workers’ Perspective]. Gut Geschützt im Betrieb? Arbeitsschutz in der Corona-Pandemie aus Sicht der Beschäftigten.

[17] Rind E., Kimpel K., Preiser C., Papenfuss F., Wagner A., Alsyte K., Siegel A., Klink A., Steinhilber B., Kauderer J. (2020). Adjusting working conditions and evaluating the risk of infection during the COVID-19 pandemic in different workplace settings in Germany: A study protocol for an explorative modular mixed methods approach. BMJ Open.

[18] Sears J.M., Wickizer T.M., Franklin G.M., Fulton-Kehoe D., Hannon P.A., Harris J.R., Graves J.M., McGovern P.M. (2023). Development and maturation of the occupational health services research field in the United States over the past 25 years: Challenges and opportunities for the future. Am. J. Ind. Med..

[19] Ansmann L., Nöst S., Körner M., Auschra C., Bal R., Böddekder M., Bode I., Braithwaite J., Breidenbach C., Coors M. (2024). Navigating the Future of Organisational Health Services Research in Germany and beyond: A position paper. Gesundheitswesen.

[20] Forum Gesundheitsstandort Baden-Württemberg [Occupational Health Care Research: Supporting Companies in the Implementation and Acceptance of Infection Control and Changed Working Conditions in the Context of the COVID 19 Pandemic]. Arbeitsmedizinische Versorgungsforschung: Unterstützung von Firmen mit Blick auf die Umsetzung und Akzeptanz des Infektionsschutzes und Geänderter Arbeitsbedingungen im Rahmen der COVID-19-Pandemie. https://www.forum-gesundheitsstandort-bw.de/projekte/ministerium-fuer-wissenschaft-forschung-und-kunst-2/begleitung-von-firmen-waehrend-der-wiederaufnahme-der-produktion-bzw-von-taetigkeiten-vor-ort-mit-blick-auf-die-umsetzung-und-ak.

[21] Skivington K., Matthews L., Simpson S.A., Craig P., Baird J., Blazeby J.M., Boyd K.A., Craig N., French D.P., McIntosh E. (2021). A new framework for developing and evaluating complex interventions: Update of Medical Research Council guidance. BMJ.

[22] STROBE Checklist for Cross-Sectional Studies. https://www.strobe-statement.org/checklists/.

[23] Federal Ministry of Labor and Social Affairs (BMAS) [SARS-CoV-2 Occupational Health and Safety Ordinance, 25 June 2021]. SARS-CoV-2-Arbeitsschutzverordnung (Corona-ArbSchV) vom 25. Juni 2021. https://www.bundesanzeiger.de/pub/publication/sDQKZQJfie80yIKIp5W/content/sDQKZQJfie80yIKIp5W/BAnz%20AT%2028.06.2021%20V1.pdf?inline.

[24] Council of the European Communities (2003). Commission Recommendation of 6 May 2003 Concerning the Definition of Micro, Small and Medium-Sized Enterprises. Off. J. Eur. Union.

[25] Stratton S.J. (2021). Population Research: Convenience Sampling Strategies. Prehospital Disaster Med..

[26] Unipark Online Umfrage [Online Survey]. https://www.unipark.com/.

[27] OCR System GmbH FormPro—Scan and Capture Data from Questionnaires, Surveys and Forms. https://www.ocr-systeme.de/en/index/formpro/FormPro.

[28] Soeder J., Neunhöffer A.T., Wagner A., Preiser C., Rebholz B., Montano D., Schmitz N., Kauderer J., Papenfuss F., Klink A. (2022). Assessing Differences in Attitudes toward Occupational Safety and Health Measures for Infection Control between Office and Assembly Line Employees during the COVID-19 Pandemic in Germany: A Cross-Sectional Analysis of Baseline Data from a Repeated Employee Survey. Int. J. Environ. Res. Public Health.

[29] Beck D., Lenhardt U. (2019). Consideration of psychosocial factors in workplace risk assessments: Findings from a company survey in Germany. Int. Arch. Occup. Environ. Health.

[30] Beckmann K., Glemser A., Heckel C., von der Heyde C., Hoffmeyer-Zlotnik J., Hanefeld U., Herter-Eschweiler R., Kühnen C. (2010). [Demographic Standards: A Joint Recommendation of the ADM, the ASI and the Federal Statistical Office]. Demographische Standards: Eine gemeinsame Empfehlung des ADM Arbeitskreis Deutscher Markt- und Sozialforschungsinstitute e.V., der Arbeitsgemeinschaft Sozialwissenschaftlicher Institute e.V. (ASI) und des Statistischen Bundesamtes.

[31] Berling I., Jollenbeck M., Stamer T., Ochsmann E. (2022). Association between mobile work and work ability: A longitudinal study under the impact of the COVID-19 pandemic. Int. Arch. Occup. Environ. Health.

[32] Betsch C., Wieler L., Bosnjak M., Ramharter M., Stollorz V., Omer S., Korn L., Sprengholz P., Felgendreff L., Eitze S. (2020). COSMO—COVID-19 Snapshot Monitoring (COSMO Standard): Monitoring Knowledge, Risk Perceptions, Preventive Behaviours, and Public Trust in the Current Coronavirus Outbreak: Fragebögen: [Questionnaires—COSMO Open]. https://dfncloud.uni-erfurt.de/s/Cmzfw8fPRAgzEpA.

[33] Gesis Metadata for Official Statistics—Questionnaires: MZ. [Microcensus 2018 Questionnaire]. https://www.gesis.org/en/missy/materials/MZ/documents/frageboegen.

[34] Rammstedt B., Kemper C., Klein M., Beierlein C., Kovaleva A. (2012). [A Brief Scale for Measuring the Five Dimensions of Personality: Big Five Inventory-10 (BFI-10)]. Eine kurze Skala zur Messung der fünf Dimensionen der Persönlichkeit: Big-Five-Inventory-10 (BFI-10).

[35] Nübling M., Stößel U., Hasselhorn H.-M., Michaelis M., Hofman F. (2006). Measuring psychological stress and strain at work: Evaluation of the COPSOQ Questionnaire in Germany. Psycho-Soc.-Med..

[36] Ochsmann E. [Longitudinal Study onWork and Health Under the Impact of the COVID-19 Pandemic]. Längsschnittstudie zu Arbeit und Gesundheit in Zeiten der Corona-Pandemie. https://www.uksh.de/arbeitsmedizin-luebeck/Forschung/Forschungsprojekte.html.

[37] Rammstedt B., Kemper C., Klein M., Beierlein C., Kovaleva A. Big Five Inventory (BFI-10) 2014. https://zis.gesis.org/skala/Rammstedt-Kemper-Klein-Beierlein-Kovaleva-Big-Five-Inventory-(BFI-10).

[38] Sommer S., Schmitt-Howe B. [Company and Employee Survey 2015 as Part of the Evaluation of the Joint German Occupational Safety and Health Strategy]. Betriebs- und Beschäftigtenbefragung 2015 im Rahmen der Dachevaluation der Gemeinsamen Deutschen Arbeitsschutzstrategie. https://search.gesis.org/research_data/ZA6759.

[39] Wagner A., Rieger M.A., Manser T., Sturm H., Hardt J., Martus P., Lessing C., Hammer A., WorkSafeMed C. (2019). Healthcare professionals’ perspectives on working conditions, leadership, and safety climate: A cross-sectional study. BMC Health Serv. Res..

[40] WHO Regional Office for Europe (2018). COVID-19 Snapshot Monitoring (COSMO Standard): Monitoring Knowledge, Risk Perceptions, Preventive Behaviours, and Public Trust in the Current Coronavirus Outbreak—WHO Standard Protocol. https://www.psycharchives.org/en/item/62216bdb-69fa-44e7-92b4-8438b3817341.

[41] Kemper C., Beierlein C., Bensch D., Kovaleva A., Rammstedt B. (2012). [A Short Scale for Measuring the Gamma Factor of Socially Desirable Response Behavior The Social Desirability Gamma Short Scale (KSE-G)]. Eine Kurzskala zur Erfassung des Gamma-Faktors sozial erwünschten Antwortverhaltens: Die Kurzskala Soziale Erwünschtheit-Gamma (KSE-G).

[42] Lechert Y., Schroedter J., Lüttinger P. [The CASMIN Education Classification: The Implementation of the CASMIN Education Classification for the 1970 Census, the 1971 Microcensus Supplementary Survey and the 1976–2004 Microcensuses]. Die Bildungsklassifikation CASMIN: Die Umsetzung der Bildungsklassifikation CASMIN für die Volkszählung 1970, die Mikrozensus-Zusatzerhebung 1971 und die Mikrozensen 1976–2004. https://nbn-resolving.org/urn:nbn:de:0168-ssoar-262353.

[43] Robert Koch-Institut (RKI) (2020). [Coronavirus SARS-CoV-2—Information and Guidance for People at Increased Risk of Severe Disease Progression COVID-19: Groups of People Known to be at Increased Risk of Severe Disease Progression 2020]. Coronavirus SARS-CoV-2—Informationen und Hilfestellungen für Personen mit Einem Höheren Risiko für Einen Schweren COVID-19—Krankheitsverlauf: Personengruppen, die Nach Bisherigen Erkenntnissen ein Höheres Risiko für Einen Schweren Krankheitsverlauf Haben. https://www.rki.de/DE/Content/InfAZ/N/Neuartiges_Coronavirus/Risikogruppen.html.

[44] Newman D.A. (2014). Missing Data. Organ. Res. Methods.

[45] Bühner M., Zielger M. (2009). Statistik für Psychologen und Sozialwissenschaftler. [Statistics for Psychologists and Social Scientists].

[46] Cohen J. (1988). Statistical Power Analysis for the Behavioral Sciences.

[47] Fey C.F., Hu T., Delios A. (2022). The Measurement and Communication of Effect Sizes in Management Research. Manag. Organ. Rev..

[48] NAKO Gesundheitsstudie Fragebogen [Questionnaire]. https://nako.de/teilnahme/untersuchungen/fragebogen/.

[49] Bundesanstalt für Arbeitsschutz und Arbeitsmedizin (BAuA) (2019). [Technical Rules for Hazardous Substances—TRGS]. Technische Regeln für Gefahrstoffe. https://www.baua.de/DE/Angebote/Regelwerk/TRGS/TRGS.

[50] Khaday S., Li K.W., Dorloh H. (2023). Factors Affecting Preventive Behaviors for Safety and Health at Work during the COVID-19 Pandemic among Thai Construction Workers. Healthcare.

[51] Robelski S., Steidelmüller C., Pohlan L. (2020). [Occupational Health and Safety During the COVID-Crisis]. Betrieblicher Arbeitsschutz in der Corona-Krise.

[52] Ordysinski S. (2024). The role of trust in occupational safety: Research results. Int. J. Occup. Saf. Ergon..

[53] Sarracino F., Greyling T., O’Connor K.J., Peroni C., Rossouw S. (2024). Trust predicts compliance with COVID-19 containment policies: Evidence from ten countries using big data. Econ. Hum. Biol..

[54] COSMO COVID-19 Snapshot Monitoring. Reactance: July–August 2021. https://projekte.uni-erfurt.de/cosmo2020/web/explorer/.

[55] Bokhari R., Shahzad S. (2022). Explaining Resistance to the COVID-19 Preventive Measures: A Psychological Reactance Perspective. Sustainability.

[56] Wagner A., Rind E., Burgess S., Bockelmann I., Thielmann B., Heinz H., Siegel A., Schroder V., Jockel K.H., Husing A. (2025). Shaping a positive occupational safety climate in general practice teams-findings of the baseline survey of the cluster randomized IMPROVE *job* trial. Front. Public Health.

[57] Wagner A., Schöne L., Rieger M.A. (2020). Determinants of Occupational Safety Culture in Hospitals and other Workplaces-Results from an Integrative Literature Review. Int. J. Environ. Res. Public Health.

[58] Reuter M., Rigo M., Formazin M., Liebers F., Latza U., Castell S., Jockel K.H., Greiser K.H., Michels K.B., Krause G. (2022). Occupation and SARS-CoV-2 infection risk among 108 960 workers during the first pandemic wave in Germany. Scand. J. Work Environ. Health.

[59] Romero Starke K., Mauer R., Hegewald J., Bolm-Audorff U., Bruckner G., Schussel K., Schroder H., Seidler A. (2024). Occupational risks of COVID-19: A case-cohort study using health insurance claims data in Germany. BMC Public Health.

[60] Wahrendorf M., Schaps V., Reuter M., Hoebel J., Wachtler B., Jacob J., Alibone M., Dragano N. (2023). Occupational differences of COVID-19 morbidity and mortality in Germany. An analysis of health insurance data from 3.17 million insured persons. Bundesgesundheitsblatt Gesundheitsforschung Gesundheitsschutz.

[61] Gabriel K.M.A., Schroder C., Wolf R., Bolm-Audorff U., Kienast C., Smolinska J., Petereit-Haack G., Seidler A. (2025). SARS-CoV-2 infection risk by non-healthcare occupations: A systematic review and meta-analysis. J. Occup. Med. Toxicol..

[62] Bogoeski V. (2022). Continuities of exploitation: Seasonal migrant workers in German agriculture during the COVID-19 pandemic. J. Law Soc..

[63] Charlton D. (2022). Seasonal farm labor and COVID-19 spread. Appl. Econ. Perspect. Policy.

[64] Küppers C. (2021). How to Harvest in a Pandemic? The German Media Coverage of Migrant Workers and Harvesting in the Context of the COVID-19 Crisis in 2020.

[65] Honan J., Ingram M., Quijada C., Chaires M., Fimbres J., Ornelas C., Sneed S., Stauber L., Spitz R., Sandoval F. (2023). Understanding the Impacts of the COVID-19 Pandemic on Small Businesses and Workers Using Quantitative and Qualitative Methods. Ann. Work Expo. Health.

[66] Couarraze S., Delamarre L., Marhar F., Quach B., Jiao J., Aviles Dorlhiac R., Saadaoui F., Liu A.S., Dubuis B., Antunes S. (2021). The major worldwide stress of healthcare professionals during the first wave of the COVID-19 pandemic—The international COVISTRESS survey. PLoS ONE.

[67] Diebig M., Gritzka S., Dragano N., Angerer P. (2021). Presentation of a participatory approach to develop preventive measures to reduce COVID-19 transmission in child care. J. Occup. Med. Toxicol..

[68] Wagner A., Werners B., Pieper C., Eilerts A.L., Seifried-Dubon T., Grot M., Junne F., Weltermann B.M., Rieger M.A., Rind E. (2023). Exploring Transfer Potentials of the IMPROVEjob Intervention for Strengthening Workplace Health Management in Micro-, Small-, and Medium-Sized Enterprises in Germany: A Qualitative Study. Int. J. Environ. Res. Public Health.

[69] Hansen A.F., Hasle P., Caroly S., Reinhold K., Jarvis M., Herrig A.O., Heiberg B.D., Sogaard K., Punnett L., Jensen Stochkendahl M. (2024). Participatory ergonomics: What works for whom and why? A realist review. Ergonomics.

[70] World Health Organization Regional Office for Europe (2008). Chapter 3: Principles for Ethical Decision-Making in Pandemic Influenza Planning. Eleventh Futures Forum on the Ethical Governance of Pandemic Influenza Preparedness.

[71] Hoven H., Dragano N., Angerer P., Apfelbacher C., Backhaus I., Hoffmann B., Icks A., Wilm S., Fangerau H., Sohner F. (2022). Striving for Health Equity: The Importance of Social Determinants of Health and Ethical Considerations in Pandemic Preparedness Planning. Int. J. Public Health.

[72] Jain A., Torres L.D., Teoh K., Leka S. (2022). The impact of national legislation on psychosocial risks on organisational action plans, psychosocial working conditions, and employee work-related stress in Europe. Soc. Sci. Med..

[73] Brenscheidt S., Siefer A., Backhaus N., Halke T., Lück M., Michels L., Vegner V. (2025). [The Changing World of Work: Figures–Data–Facts]. Arbeitswelt im Wandel: Zahlen–Daten–Fakten (2025).

[74] Schill A.L., Chosewood L.C. (2013). The NIOSH Total Worker Health™ program: An overview. J. Occup. Environ. Med..

[75] Aust B., Møller J.L., Nordentoft M., Frydendall K.B., Bengtsen E., Jensen A.B., Garde A.H., Kompier M., Semmer N., Rugulies R. (2023). How effective are organizational-level interventions in improving the psychosocial work environment, health, and retention of workers? A systematic overview of systematic reviews. Scand. J. Work Environ. Health.

[76] Aust B., Leduc C., Cresswell-Smith J., O’Brien C., Rugulies R., Leduc M., Dhalaigh D.N., Dushaj A., Fanaj N., Guinart D. (2024). The effects of different types of organisational workplace mental health interventions on mental health and wellbeing in healthcare workers: A systematic review. Int. Arch. Occup. Environ. Health.

[77] Neunhoffer A.T., Gibilaro J., Wagner A., Soeder J., Rebholz B., Blumenstock G., Martus P., Rieger M.A., Rind E. (2022). Factors Associated with the COVID-19 Vaccination Status of Higher Education Students: Results of an Online Cross-Sectional Survey at Six Universities in Southwestern Germany. Vaccines.

[78] Rind E., Ehmann A., Wagner A., Soeder J., Preiser C., Rieger M.A. (2022). Immunization coverage is essential for teaching and learning on campus during the COVID-19 pandemic. Saf. Health Work.

[79] Soeder J., Preiser C., Wagner A., Neunhöffer A.T., Papenfuss F., Schwille-Kiuntke J., Wittich A., Rind E., Rieger M.A. (2025). Pandemic preparedness in shaping psychosocial working conditions—Insights for occupational safety and health from a longitudinal mixed-methods study during the COVID-19 pandemic at six company sites of one organization in Germany. PLoS ONE.

[80] Soeder J., Wagner A., Neunhoffer A.T., Martus P., Papenfuss F., Wittich A., Schwille-Kiuntke J., Rind E., Rieger M.A. (2024). Exploring organizational aspects that promote health-related preventive behavior—Using the example of work-related SARS-CoV-2-infection control measures in Germany, August 2020 to November 2021. Front. Public Health.

[81] Wagner A., Keles K., Preiser C., Neunhöffer A.T., Soeder J., Schwille-Kiuntke J., Rieger M.A., Rind E. (2023). Assessing Attitudes and Participation Regarding a Pilot COVID-19 Workplace Vaccination Program in Southern Germany Considering the Occupational Health Perspective-A Mixed Methods Study. Vaccines.

[82] DESTATIS [Classification of Economic Activities, 2008 Edition (WZ 2008)]. Klassifikation der Wirtschaftszweige, Ausgabe 2008 (WZ 2008). https://www.destatis.de/DE/Methoden/Klassifikationen/Gueter-Wirtschaftsklassifikationen/klassifikation-wz-2008.html.

[83] German Social Accident Insurance (DGUV) Companies by Size in 2023. Accident Insurance in Industrial Sector. https://www.dguv.de/en/facts-figures/insured-person-companies/company-size/index.jsp.

[84] Völter-Mahlknecht S., Michaelis M., Preiser C., Blomberg N., Rieger M. (2011). Forschungsbericht 448. Inanspruchnahme von Angebotsuntersuchungen in der arbeitsmedizinischen Vorsorge [Research Report 448. Use of optional examinations in occupational health prevention].

[85] Freiburger Forschungsstelle für Arbeitswissenschaften GmbH (FFAW) (2020). COPSOQ—Die Befragung zu Psychischen Belastungen am Arbeitsplatz [COPSOQ-Questionnaire About Work-Related Psychosocial Demands]. https://www.copsoq.de/kontakt/.

