# Supplementary material for: Investigating Factors Associated with Employees’ Attitudes Towards Work-Related Infection Control Measures During the COVID-19 Pandemic: An Exploratory Cross-Sectional Study from Seven Different Companies in Germany, July–August 2021
Source: Healthcare (Basel). 2025 Sep 27;13(19):2454. doi: 10.3390/healthcare13192454 (PMC12524139; doi:10.3390/healthcare13192454)
Supplement: Supplementary file 1 [file healthcare-13-02454-s001.zip › healthcare-3835815-supplementary.pdf]

Investigating Factors Associated with Employees' Attitudes Towards Work-Related Infection Control Measures During the COVID-19 Pandemic: An Exploratory Cross-Sectional Study from Seven Different Companies in Germany, July–August 2021

The ***study protocol*** can be downloaded here: https://bmjopen.bmj.com/content/10/11/e043908

(Doi: 10.1136/bmjopen-2020-043908; last access: 7 August 2025)

Other studies related to this article, please see references [1–5].

Table S1. STROBE-checklist for cross-sectional studies

Table S2. Components of the employee survey

Table S3. Potential predictors of the main outcome 'attitudes towards the suitability of work-related SARS-CoV-2 infection control measures'

Table S4. Response rate of the participating companies (n = 7) by industry sector and type of affiliated statutory accident insurance

Table S5. Results of the bivariate linear regression analysis

Supplementary Table S1. STROBE-checklist for cross-sectional studies [6]

	Item No	Recommendation	Reported on page #
**Title and abstract**	1	(*a*) Indicate the study's design with a commonly used term in the title or the abstract	1
		(*b*) Provide in the abstract an informative and balanced summary of what was done and what was found	1
**Introduction**			
Background/ rationale	2	Explain the scientific background and rationale for the investigation being reported	2-3
Objectives	3	State specific objectives, including any prespecified hypotheses	3, 11
**Methods**			
Study design	4	Present key elements of study design early in the paper	3
Setting	5	Describe the setting, locations, and relevant dates, including periods of recruitment, exposure, follow-up, and data collection	4-6
Participants	6	(*a*) Give the eligibility criteria, and the sources and methods of selection of participants	4
Variables	7	Clearly define all outcomes, exposures, predictors, potential confounders, and effect modifiers. Give diagnostic criteria, if applicable	4-6
Data sources/ measurement	8*	For each variable of interest, give sources of data and details of methods of assessment (measurement). Describe comparability of assessment methods if there is more than one group	4-6, S2
Bias	9	Describe any efforts to address potential sources of bias	4, 13
Study size	10	Explain how the study size was arrived at	4, 6
Quantitative variables	11	Explain how quantitative variables were handled in the analyses. If applicable, describe which groupings were chosen and why	4-6
Statistical methods	12	(*a*) Describe all statistical methods, including those used to control for confounding	5, 6
		(*b*) Describe any methods used to examine subgroups and interactions	5-6
		(*c*) Explain how missing data were addressed	5
		(*d*) If applicable, describe analytical methods taking account of sampling strategy	n/a
		(*e*) Describe any sensitivity analyses	n/a
**Results**			
Participants	13*	(a) Report numbers of individuals at each stage of study—e.g. numbers potentially eligible, examined for eligibility, confirmed eligible, included in the study, completing follow-up, and analyzed	6-7
		(b) Give reasons for non-participation at each stage	
		(c) Consider use of a flow diagram	
Descriptive data	14*	(a) Give characteristics of study participants (e.g. demographic, clinical, social) and information on exposures and potential confounders	

	Item No	Recommendation	Reported on page #
		(b) Indicate number of participants with missing data for each variable of interest	6, Table 1
Outcome data	15*	Report numbers of outcome events or summary measures	6-11
Main results	16	(*a*) Give unadjusted estimates and, if applicable, confounder-adjusted estimates and their precision (e.g., 95% confidence interval). Make clear which confounders were adjusted for and why they were included	6-8, S3, S5
		(*b*) Report category boundaries when continuous variables were categorized	S3
		(*c*) If relevant, consider translating estimates of relative risk into absolute risk for a meaningful time period	n/a
Other analyses	17	Report other analyses done—e.g. analyses of subgroups and interactions, and sensitivity analyses	9
**Discussion**			
Key results	18	Summarize key results with reference to study objectives	11-13
Limitations	19	Discuss limitations of the study, taking into account sources of potential bias or imprecision. Discuss both direction and magnitude of any potential bias	13-14
Interpretation	20	Give a cautious overall interpretation of results considering objectives, limitations, multiplicity of analyses, results from similar studies, and other relevant evidence	14-15
Generalizability	21	Discuss the generalizability (external validity) of the study results	14, 15
**Other information**			
Funding	22	Give the source of funding and the role of the funders for the present study and, if applicable, for the original study on which the present article is based	16

*Give information separately for exposed and unexposed groups.

Note: An Explanation and Elaboration article discusses each checklist item and gives methodological background and published examples of transparent reporting. The STROBE checklist is best used in conjunction with this article (freely available on the Web sites of PLoS Medicine at http://www.plosmedicine.org/, Annals of Internal Medicine at http://www.annals.org/, and Epidemiology at http://www.epidem.com/). Information on the STROBE Initiative is available at www.strobe-statement.org.

Table S2. Components of the employee survey according to BMAS 2020 [7] and Söder et al. 2022 [8, Supplementary File S2]

Variables / Scores	Category/ Scale	Items	Source
**Outcome / subscores**			
Attitude towards recommended **technical** safety and health measures for infection control	10 questions on a 5-point Likert Scale	How appropriate do you consider the following recommended measures to prevent the spread and infection of the novel coronavirus in the workplace? Please evaluate all preventive measures on a technical level listed below.<br><br>Not appropriate at all ... very appropriate<br><br>1. Keep distance from other people / Maintain a distance of at least 1.5 m at the workplace<br>2. Ventilate rooms regularly<br>3. Protection of employees with, for example, plexiglass planes<br>4. A distance of at least 1.5 m between the individual workstations must be maintained, and workplaces should be arranged accordingly<br>5. Form fixed teams in order to reduce mixing<br>6. Home office, if possible<br>7. Meetings are preferably held online/ via Skype<br>8. Use large rooms to ensure a distance of 1.5 m<br>9. Reduce spent time at the canteen or on-site stores, especially during peak hours<br>10. Perform frequent cleaning of offices, meeting rooms, kitchenettes, traffic routes, sanitary facilities, etc.	Self-developed item based on [7]
Attitude towards recommended **organisational** safety and health measures for infection control	6 questions on a 5-point Likert Scale	How appropriate do you consider the following recommended measures to prevent the spread and infection of the novel coronavirus in the workplace? Please evaluate all preventive measures on a technical level listed below.<br><br>Not appropriate at all ... very appropriate<br><br>1. Define personally assigned workstations, if possible<br>2. Define personally assigned work equipment (e.g., mouse/ keyboard)<br>3. Avoid group gatherings (e.g., shift information transfer via electronic media, where possible)	Self-developed item based on [7]

Variables / Scores	Category/ Scale	Items	Source
**Outcome / subscores**			
		4. Avoid unnecessary through traffic in highly frequented areas (e.g., offices, halls)<br>5. Break times are decoupled<br>6. Supervision of compliance with the hygiene rules by supervisor	
Attitude towards recommended **personal** safety and health measures for infection control	10 questions on a 5-point Likert Scale	How appropriate do you consider the following recommended measures to prevent the spread and infection of the novel coronavirus in the workplace? Please evaluate all preventive measures on a technical level listed below.<br><br>Not appropriate at all ... very appropriate<br><br>1. Wash hands thoroughly with soap and water regularly<br>2. Avoid personal greetings by shaking hands / avoid shaking hands<br>3. Cough into your sleeve and turn away from others when coughing<br>4. Mouth-nose-covers (MNC) must be worn<br>5. Employees with cold symptoms and/ or fever are to stay at home<br>6. Preferably use your personal vehicle or other personal form of transportation<br>7. Special measures to protect employees at high risk are introduced<br>8. Consulting by the on-site doctor / occupational physician<br>9. Install informational signs covering hygiene and rules of conduct at the workplace<br>10. A communication guide for employees has been prepared (hygiene and conduct rules) and posted in highly frequented areas	Self-developed item based on [7]

Table S3. Potential predictors of the main outcome 'attitudes towards the suitability of work-related SARS-CoV-2 infection control measures' [N = 821]

Variable [Reference]	Mean (Standard Deviation) or percentages	[n_{valid}]
**I. Socio-demographic characteristics** (including Big Five personality traits and social desirability)		
Age (years) [9,10]		**[679]**
18 - 29	16.1%	[109]
30 – 49	46.4%	[315]
> 49	37.6%	[255]
Gender [9,10]		**[791]**
male	56.0%	[443]
female (Ref+)	43.2%	[342]
diverse*	0.8%	[6]
Country of birth [11] †		**[789]**
Germany	90.6%	[715]
other country (Ref+)	8.6%	[68]
do not know*	0.8%	[6]
Education [12]		**[804]**
primary	6.1%	[49]
intermediate	24.8%	[199]
higher (Ref+)	69.1%	[556]
Living in a committed relationship [9,10] †		**[771]**
no (Ref+)	17.8%	[137]
yes	82.2%	[634]
Household size [9,10] †		**[794]**
alone (Ref+)	14.2%	[113]
at least 2 persons	85.7%	[681]
Children < 18 years living in the household		**[784]**
no (Ref+)	35.2%	[508]
yes	64.8%	[276]
Health professional in the household [9,10] †		**[790]**
no (Ref+)	89.1%	[704]
yes	10.9%	[86]
Big Five personality traits [13] (5-point Likert scale from "low" to "high")		
personality trait extraversion	3.3 (0.9)	[800]
personality trait neuroticism	2.7 (0.9)	[800]
personality trait openness	3.3 (0.9)	[800]
personality trait agreeableness	3.2 (0.8)	[800]
personality trait conscientiousness	4.0 (0.7)	[800]
Social Desirability [14]		

Variable [Reference]	Mean (Standard Deviation) or percentages	[n_{valid}]
(5-point Likert scale from "not at all" to "very much")		
social desirability of exaggerating positive qualities	1.4 (0.5)	[794]
social desirability of exaggerating negative qualities	1.9 (0.6)	[798]
**II. General workplace characteristics** (company characteristics and job-related characteristics)		
Company size** [15]		**[821]**
medium-sized company (50-249 employees) (Ref+)	6.8%	[56]
large company (≥ 250 employees)	93.2%	[765]
Industry sector [based on 16]		**[821]**
Production industry (furniture, automotive, textile, synthetic materials)	81.7%	[671]
Logistics	6.6%	[54]
Chemical and water industry (Ref+)	11.7%	[96]
Affiliated statutory accident insurance [17]		**[821]**
Trade and logistics industry	5.0%	[41]
Energy, textile, electrical and media products	18.0%	[148]
Transport industry, postal logistics and telecommunications	6.6%	[54]
Raw materials and chemical industry	6.7%	[55]
Woodworking and metalworking industries (Ref+)	63.7%	[523]
Employment at company (years) [18] †	3.0 (11.0)	[806]
Work in changing teams		**[810]**
no (Ref+)	77.7%	[629]
yes	22.3%	[181]
Shift work		**[813]**
no (Ref+)	93.5%	[760]
yes	6.5%	[53]
Short-time work		**[811]**
no (Ref+)	89.3%	[724]
yes	10.7%	[87]
Full-time job [19]		**[815]**
no (Ref+)	16.0%	[130]
yes	84.0%	[685]
Fixed-term contract [19]		**[813]**
no (Ref+)	91.1%	[741]
yes	8.9%	[72]
Leadership position [19]		**[814]**
no (Ref+)	76.5%	[623]
Yes	23.5%	[191]
Professional activity		**[815]**
assembly line/production/warehouse	17.7%	[144]

Variable [Reference]	Mean (Standard Deviation) or percentages	[n_{valid}]
office on-site	53.7%	[438]
office remote work (Ref+)	28.6%	[233]
**III. Workplace characteristics related to the COVID-19 pandemic**		
Perceived psychosocial demands [19–21] † (5-point Likert scale from “negative” to “positive” rating)		
perceived psychosocial demands from aspects of work content at work during the pandemic	3.4 (0.8)	[813]
perceived psychosocial demands from aspects of the work organisation during the pandemic	2.5 (0.9)	[812]
perceived psychosocial demands from aspects of work environment during the pandemic	4.0 (1.0)	[815]
perceived psychosocial demands from aspects of social relations during the pandemic	3.8 (0.9)	[818]
[1] Trust in colleagues’ adherence to distance and hygiene rules [9,10]† (7-point Likert scale from “not at all” to “always”)	5.2 (1.5)	[816]
Perceived probability of contracting COVID-19 in the workplace [9,10]† (7-point Likert scale from “extremely unlikely” to “extremely likely”)	3.6 (1.7)	[817]
Perceived self-efficacy to avoid a SARS-CoV-2 infection in the current workplace situation [9,10]† (7-point Likert scale from “extremely difficult” to “extremely easy”)	5.0 (1.5)	[811]
Feeling informed about possible risks of SARS-CoV-2 infection at work [22]†		**[801]**
poor/satisfactory (Ref+)	21.1%	[169]
good/very good	78.9%	[632]
[2] COVID-19 specific reactance [9,10]† (7-point Likert scale from “not at all” to “very much”)	3.2 (1.8)	[817]
Leadership quality as rated by employees [19] (1 = lowest rating to 100 = highest rating)	55.8 (24.6)	[788]
Employees’ rating of the employer’s commitment to occupational safety and health related to SARS-CoV-2 [22]† (4-point Likert scale from “very low” to “very high”)	3.2 (0.8)	[789]
**IV. Perception of SARS-CoV-2 and COVID-19 in general**		
[3] Disease perception [9,10] (7-point Likert scale from “low” to “high”)	5.2 (1.3)	[810]
[4] Affective risk perception [9,10] (7-point Likert scale from “low” to “high”)	4.4 (1.3)	[814]
Perceived adequacy of SARS-CoV-2 media coverage [9,10] (7-point Likert scale from “not media hyped” to “media hyped”)	5.1 (1.3)	[811]
Frequency of self-information regarding SARS-CoV-2 / COVID-19 [9,10] (7-point Likert scale from “never” to “very often”)	5.1 (1.6)	[815]
[5] Perceived personal susceptibility and expected severity of infection with SARS-CoV-2 in private surroundings [9,10]† (7-point Likert scaled from lowest to highest)	3.7 (1.1)	[816]
[6] Affiliation to risk group for developing severe COVID-19 [23]		**[770]**
no/not specified (Ref+)	80.8%	[242]

Variable [Reference]	Mean (Standard Deviation) or percentages [n_{valid}]	
yes	19.2%	[537]
[7] Frequent contact with individuals from the risk group		**[779]**
no (Ref+)	31.1%	[242]
yes	68.9%	[537]
(Inter)national travelling and recreational activities with an increased risk of infection		**[796]**
no (Ref+)	59.7%	[475]
yes	40.3%	[321]
[8] Knowledge about confirmed cases of infected peers† [9,10]		**[783]**
no (Ref+)	20.2%	[158]
yes	79.8%	[625]
[9] COVID-19- specific resilience [9,10] (7-point Likert scale from "low" to "high")	5.3 (1.2)	[813]
**V. Variables relating to SARS-CoV-2 rapid antigen testing and COVID-19 vaccination**		
Potential readiness to perform SARS-CoV-2 rapid antigen tests† [9,10] (7-point Likert scale from "low" to "high")	5.7 (1.8)	[814]
Rapid antigen test ever performed or performed in the last two weeks		**[811]**
no (Ref+)	26.9%	
yes	73.1%	
Use of the rapid antigen tests provided by the company	27,7%	[216]
no (Ref+)	72,3%	[565]
yes		
Positive rapid antigen test result ever obtained [9,10]	98.1%	**[783]**
no (Ref+)	1.9%	[15]
yes		
[10] Perceived benefit of COVID-19 vaccination for the individual or the company [self-developed based on 9, 10] (7-point Likert scale from "low" to "high")	5.2 (1.4)	[816]
Potential readiness for COVID-19 vaccination within the next week [9,10] (7-point Likert scale from "not at all" to "high")	6.2 (1.8)	[806]
One or two vaccination doses received [9,10]		**[807]**
no (Ref+)	27.7%	[100]
yes	87.6%	[707]

Note: Translated to English by the authors for the purpose of publication. The original version was in German only. See Söder et al. 2022: supplementary file S2 [8].
+ Reference group
† Adapted to the target group
* Category was excluded from further analysis because n < 10
**We could not recruit small-sized companies

[1] Two items: I trust my colleagues to 1. adhere to the distance rules in the workplace and 2. adhere to the hygiene rules in the workplace

[2] Four items: 1. Do you perceive the measures as restricting your freedom? 2. How disturbed do you feel by the measures? 3. How frustrated do you feel about the measures? 4. How annoyed do you feel about the measures?

[3] Two items: the novel virus to me feels 1. far away from me - close to me and 2. spreading slowly - spreading fast

[4] Three items: the novel virus is - 1. something I almost never think about - something I think about all the time 2. not fear-inducing - fear-inducing 3. not worrying – worrying

[5] Three items: 1. perceived personal susceptibility, 2. expected severity of infection with SARS-CoV-2, 3. perceived probability of contracting COVID-19 in private surrounding

[6] An increased risk is assumed for individuals with coronary heart disease, diabetes mellitus, bronchial asthma or chronic bronchitis, liver disease, kidney problems, cancer, and diseases associated with immunodeficiency

[7] Three multiple choice answers: 1. due to age and/or pre-existing diseases, 2. in nursing homes, hospitals, etc., 3. cared for by outpatient care service (at least one item = yes)

[8] Four multiple choice answers: yes … 1. suspected but not yet confirmed by a test, 2. confirmed/recovered from infection, 3. deceased due to infection, 4. no/don't know

[9] Related to own experiences in the pandemic, please indicate to what extent you agree with each statement: 1. I find ways to keep going, 2. I know that I can get through hard times, 3… I learn important lessons for life, 4. I am learning ways to cope better next time (1-7; strongly disagree - strongly agree)

[10] Three items: Vaccinating against COVID-19… 1. mainly helps to preserve my health, 2. mainly helps to eliminate the disadvantages I have due to the pandemic, e.g. contact reduction and lockdown, 3. mainly helps my employer to eliminate the disadvantages I have due to the pandemic (1-7; disagree completely - agree completely)

Table S4. Response rate of the participating companies (n = 7) by industry sector and type of affiliated statutory accident insurance

**Company number: industry sector**	**Type of affiliated social accident insurance**	**Response rate in %[1] (n participants/employees addressed)**
1: Chemical & pharmaceutical industry	Trade & logistics industry	33.1% (41/124)
2: Assembly line & production industry	Woodworking & metalworking industries	42.6% (46/108)
3: Assembly line & production industry	Woodworking & metalworking industries	12.8% (10/78)
4: Assembly line & production industry [2]	Woodworking & metalworking industries	23.7% & 16.4% (360/1516 & 107/654)
5: Assembly line & production industry	Energy, textile, electrical & media products sectors	31.2% (148/475)
6: Logistics	Commercial transport, postal logistics & telecommunication	12.0% (54/450)
7: Chemical & pharmaceutical industry	Raw materials & chemical industry	24.2% (55/227)

[1] Average total response rate 24.5% (SD 10.7%; range 12.0% - 42.6%)

[2] Two company sites

Table S5. Results of the bivariate linear regression analysis: effects of possible predictors for the outcome 'attitudes towards the suitability of work-related SARS-CoV-2 infection control measures' (wICM, total score) and subscores regarding technical (T), organisational (O), or personal (P) measures **(**imputed data; N = 821**)**

	**β (*p*) of total score (wICM) and subscores (T – O – P)**[1]			
**Variable**	**wICM**[1] (total score)	Technical measures	Organisational measures	Personal measures
**I. Sociodemographic characteristics (including Big Five personality traits and social desirability)**				
Gender (0= female and 1 = male)	-0.18 (<.001)	-0.16 (<.001)	-0.16 (<.001)	-0.17 (<.001)
Country of birth (0 = not Germany and 1 = Germany)	0.00 (.944)	-0.03 (.319)	-0.02 (.625)	0.05 (.185)
Education (Ref[2]: higher):				
primary (0 = no and 1 = yes)	-0.01 (.699)	-0.07 (.047)	-0.04 (.217)	0.08 (.019)
intermediate (ditto)	-0.03 (.927)	-0.19 (.584)	-0.03 (.354)	0.04 (.203)
Living in a committed relationship (0 = no and 1 = yes)	0.04 (.200)	0.01 (.705)	0.06 (.079)	0.05 (.118)
Household size (0 = alone and 1 = at least 2 persons)	-0.00 (.943)	-0.01 (.721)	-0.00 (.853)	-0.03 (.427)
Children < 18 years living in the household (0 = no and 1 = yes)	0.04 (.312)	0.04 (.293)	0.05 (.154)	0.01 (.795)
Health professional in household (0 = no and 1 = yes)	-0.05 (.153)	-0.06 (.107)	-0.05 (.131)	-0.02 (.475)
Big 5 personality trait extraversion (1 = low to 5 = high)	0.03 (.342)	0.03 (.362)	0.02 (.557)	0.04 (.314)
Big 5 personality trait neuroticism (ditto)	0.05 (.170)	0.02 (.520)	0.07 (.051)	0.04 (.199)
Big 5 personality trait openness (ditto)	0.09 (.008)	0.05 (.037)	0.05 (.136)	0.12 (<.001)
Big 5 personality trait agreeableness (ditto)	0.05 (.174)	0.04 (.313)	0.05 (.166)	0.05 (.173)
Big 5 personality trait conscientiousness (ditto)	0.08 (.021)	0.06 (.109)	0.05 (.133)	0.11 (.002)
Social desirability of exaggerating positive qualities (1 = not at all to 5 = very much)	0.00 (.897)	0.00 (.981)	0.02 (.549)	-0.01 (.860)
Social desirability of exaggerating negative qualities (ditto)	0.05 (.137)	0.05 (.152)	0.04 (.234)	0.05 (.180)

**II. General workplace characteristics (company characteristics and job-related characteristics)**				
Company size (0 = medium and 1 = large)	-0.01 (.735)	0.03 (.472)	0.00 (.983)	-0.06 (.080)
Industry sector (Ref[2]: Chemical and waters industry):				
Production industry (0 = no and 1 = yes)	0.09 (.008)	0.09 (.007)	0.09 (.007)	0.06 (.069)
Logistics (ditto)	-0.01 (.870)	-0.02 (.584)	0.01 (.792)	-0.00 (.986)
Affiliated statutory accident insurance				
(Ref: Woodworking and metalworking industries):				
Trade & logistics industry (0 = no and 1 = yes)	-0.05 (.171)	-0.03 (.331)	-0.04 (.239)	-0.06 (.113)
Energy, textile, electrical and media products sectors (ditto)	0.10 (.004)	0.09 (.014)	0.10 (.006)	0.10 (.006)
Transport industry, postal logistics and telecommunications (ditto)	-0.01 (.870)	-0.02 (.584)	0.01 (.792)	0.00 (.986)
Raw materials & chemical industry (ditto)	-0.10 (.006)	-0.10 (.005)	-0.12 (.001)	-0.05 (.158)
Employment at company (years)	0.08 (.018)	0.08 (.028)	0.04 (.258)	0.10 (.004)
Work in changing teams (0 = no and 1 = yes)	0.08 (.027)	0.11 (.003)	0.08 (.017)	0.01 (.682)
Shift work (ditto)	0.13 (<.001)	0.16 (<.001)	0.14 (<.001)	0.05 (.128)
Short-time work (ditto)	0.06 (.100)	-0.03 (.385)	-0.08 (.027)	-0.06 (.104)
Full-time job (ditto)	-0.13 (<.001)	-0.14 (<.001)	-0.12 (.001)	-0.10 (.006)
Fixed-term contract (ditto)	0.00 (.927)	0.03 (.444)	0.02 (.549)	-0.04 (.241)
Leadership position (ditto)	0.06 (.093)	0.04 (.244)	0.03 (.432)	0.09 (.011)
Professional activity (Ref[2]: office remote work)				
assembly line/production/warehouse (0 = no and 1 = yes)	-0.20 (<.001)	-0.24 (<.001)	-0.24 (<.001)	-0.05 (.168)
office on-site (ditto)	0.06 (.106)	0.06 (.103)	0.06 (.068)	0.03 (.345)

**III. Workplace characteristics related to the COVID-19 pandemic**				
Perceived psychosocial demands from				
aspects of work content at work during the pandemic (1 = negative to 5 = positive rating)	0.13 (<.001)	0.15 (<.001)	0.12 (.001)	0.06 (.079)
aspects of work organisation (ditto)	-0.01 (.806)	0.01 (.719)	-0.04 (.306)	-0.01 (.863)
aspects of work environment (ditto)	0.23 (<.001)	0.24 (<.001)	0.21 (<.001)	0.16 (<.001)
aspects of social relations during the pandemic (ditto)	0.17 (<.001)	0.13 (<.001)	0.14 (<.001)	0.20 (<.001)
Trust in colleagues' adherence to distance and hygiene rules (1 = not at all to 7 = always)	0.23 (<.001)	0.23 (<.001)	0.16 (<.001)	0.24 (<.001)
Perceived probability of contracting COVID-19 in the workplace (1 = extremely unlikely to 7 = extremely likely)	-0.05 (.156)	-0.04 (.238)	-0.02 (.584)	-0.07 (.049)
Perceived self-efficacy to avoid a SARS-CoV-2 infection in the current work-place situation (1 = extremely difficult to 7 = extremely easy)	0.19 (<.001)	0.21 (<.001)	0.13 (<.001)	0.17 (<.001)
Feeling informed about possible risks of SARS-CoV-2infection at work (0 = poor / satisfactory and 1 = good / very good)	0.24 (<.001)	0.24 (<.001)	0.18 (<.001)	0.24 (<.001)
COVID-19 specific reactance (1 = not at all to 7 = very much)	-0.35 (<.001)	-0.32 (<.001)	-0.30 (<.001)	-0.32 (<.001)
Leadership quality as rated by employees (1 = lowest to 100 = highest rating)	0.20 (<.001)	0.19 (<.001)	0.16 (<.001)	0.20 (<.001)
Employees' rating of the employer's commitment to occupational safety and health related to SARS-CoV-2 (1 = very low to 4 = very high)	0.24 (<.001)	0.24 (<.001)	0.16 (<.001)	0.24 (<.001)

**IV. Perception of SARS-CoV-2 and COVID-19 in general**				
Disease perception (1 = low to 7 = high)	0.33 (<.001)	0.28 (<.001)	0.31 (<.001)	0.32 (<.001)
Affective risk perception (ditto)	0.23 (<.001)	0.19 (<.001)	0.22 (<.001)	0.23 (<.001)
Perceived adequacy of media coverage of SARS-CoV-2 (1 = not media hyped to 7 = media hyped)	-0.21 (<.001)	-0.17 (<.001)	-0.20 (<.001)	-0.20 (<.001)
Frequency of self-information about SARS-CoV-2 (1 = never to 7 = very often)	0.19 (<.001)	0.16 (<.001)	0.16 (<.001)	0.19 (<.001)
Perceived personal susceptibility and expected severity of infection with SARS-CoV-2 in private surroundings (1 = lowest to 7 = highest)	0.19 (<.001)	0.13 (<.001)	0.19 (<.001)	0.20 (<.001)
Affiliation to risk group for developing severe COVID-19 disease (0 = no/not specified and 1 = yes)	0.04 (.238)	0.01 (.764)	0.05 (.180)	0.06 (.076)
Frequent contact with individuals from the risk group (0 = no and 1 = yes)	0.04 (.295)	0.02 (.627)	0.04 (.311)	0.05 (.143)
(Inter)national travelling and recreational activities with an increased risk of infection (ditto)	-0.01 (.694)	-0.03 (.380)	-0.01 (.879)	0.00 (.954)
Knowledge about confirmed cases of infected peers (ditto)	0.04 (.285)	0.04 (.292)	0.05 (.186)	0.02 (.664)
COVID-19- specific resilience (1 = low to 7 = high)	0.31 (<.001)	0.25 (<.001)	0.29 (<.001)	0.30 (<.001)
**V. Variables relating to SARS-CoV-2 rapid antigen testing and COVID-19 vaccination**				
Potential readiness to perform SARS-CoV-2 rapid antigen tests (ditto)	0.31 (<.001)	0.28 (<.001)	0.25 (<.001)	0.31 (<.001)
Antibody test ever performed (0 = no and 1 = yes)	0.04 (0.291)	0.03 (0.446)	0.01 (0.893)	0.06 (0.061)
Use of the rapid antigen test provided by the company (ditto)	0.15 (<.001)	0.11 (.001)	0.10 (.003)	0.17 (<.001)
Positive rapid antigen test result ever obtained (ditto)	-0.01 (.700)	0.00 (.920)	-0.01 (.732)	-0.02 (.526)
Perceived benefit of COVID-19 vaccination for the individual or the company (1 = low to 7 = high)	0.25 (<.001)	0.21 (<.001)	0.20 (<.001)	0.27 (<.001)
Potential readiness for COVID-19 vaccination within the next week (1 = not at all to 7 = high)	0.23 (<.001)	0.21 (<.001)	0.20 (<.001)	0.20 (<.001)
One or two vaccination doses received (0 = no and 1 = yes)	0.16 (<.001)	0.16 (<.001)	0.15 (<.001)	0.13 (<.001)

[1]wICM = work-related SARS-CoV-2 infection control measures (total score); orange color: $p \leq .05$; yellow color: $p<.02$; no color: $p \geq .02$. β = standardized regression coefficient beta and *p* = significance value

[2]Ref = reference category of categorical and then dichotomized variables for the subsequent multivariate regression analysis

References

1. Neunhoffer A.T.; Gibilaro J.; Wagner A.; Soeder J.; Rebholz B.; Blumenstock G.; Martus P.; Rieger M.A.; Rind E. Factors Associated with the COVID-19 Vaccination Status of Higher Education Students: Results of an Online Cross-Sectional Survey at Six Universities in Southwestern Germany. *Vaccines* 2022, *10,* 1433.
2. Rind E.; Ehmann A.; Wagner A.; Soeder J.; Preiser C.; Rieger M.A. Immunization coverage is essential for teaching and learning on campus during the COVID-19 pandemic. *Safety and Health at Work* 2022, *13,* S183-S184.
3. Soeder J.; Preiser C.; Wagner A.; Neunhöffer A.T.; Papenfuss F.; Schwille-Kiuntke J.; Wittich A.; Rind E.; Rieger M.A. Pandemic preparedness in shaping psychosocial working conditions – insights for occupational safety and health from a longitudinal mixed-methods study during the COVID-19 pandemic at six company sites of one organization in Germany. *PLOS One* 2025, *20,* e0328410.
4. Soeder J.; Wagner A.; Neunhoffer A.T.; Martus P.; Papenfuss F.; Wittich A.; Schwille-Kiuntke J.; Rind E.; Rieger M.A. Exploring organizational aspects that promote health-related preventive behavior – using the example of work-related SARS-CoV-2-infection control measures in Germany, August 2020 to November 2021. *Frontiers in Public health* 2024, *12:1388996*.
5. Wagner A.; Keles K.; Preiser C.; Neunhöffer A.T.; Soeder J.; Schwille-Kiuntke J.; Rieger M.A.; Rind E. Assessing Attitudes and Participation Regarding a Pilot COVID-19 Workplace Vaccination Program in Southern Germany Considering the Occupational Health Perspective-A Mixed Methods Study. *Vaccines* 2023, *11,* 1082.
6. STROBE. Checklist for cross-sectional studies. Available online: https://www.strobe-statement.org/checklists/ (accessed on 08.08.2024).
7. Federal Ministry of Labor and Social Affairs (BMAS). SARS-CoV-2 Occupational Safety and Health Standard (SARS-CoV-2-Arbeitsschutzstandard) - 16.04.2020. Available online: https://www.bmas.de/SharedDocs/Downloads/DE/Arbeitsschutz/sars-cov-2-arbeitsschutzstandard-en.html (accessed on 01.10.2024).
8. Soeder J.; Neunhöffer A.T.; Wagner A.; Preiser C.; Rebholz B.; Montano D.; Schmitz N.; Kauderer J.; Papenfuss F.; Klink A.; Alsyte K.; Rieger M.A.; Rind E. Assessing Differences in Attitudes toward Occupational Safety and Health Measures for Infection Control between Office and Assembly Line Employees during the COVID-19 Pandemic in Germany: A Cross-Sectional Analysis of Baseline Data from a Repeated Employee Survey. *International Journal of Environmental Research and Public Health* 2022, *20,* 614.
9. Betsch C.; Wieler L.; Bosnjak M.; Ramharter M.; Stollorz V.; Omer S.; Korn L.; Sprengholz P.; Felgendreff L.; Eitze S. COSMO - COVID-19 Snapshot Monitoring (COSMO Standard): Monitoring Knowledge, Risk Perceptions, Preventive Behaviours, and Public Trust in the Current Coronavirus Outbreak: Fragebögen: [Questionnaires—COSMO Open]. 2020. Available online: https://dfncloud.uni-erfurt.de/s/Cmzfw8fPRAgzEpA (accessed on 08.08.2024).
10. WHO Regional Office for Europe. COVID-19 Snapshot Monitoring (COSMO Standard): Monitoring Knowledge, Risk Perceptions, Preventive Behaviours, and Public Trust in the Current Coronavirus Outbreak—WHO Standard Protocol. 20.03.2018. Available online: https://www.psycharchives.org/en/item/62216bdb-69fa-44e7-92b4-8438b3817341 (accessed on 08.08.2024).
11. NAKO Gesundheitsstudie. Fragebogen [Questionnaire]. Available online: https://nako.de/teilnahme/untersuchungen/fragebogen/ (accessed on 08.10.2024).
12. Gesis. Metadata for Official Statistics—Questionnaires: MZ. [Microcensus 2018 questionnaire]. Available online: https://www.gesis.org/en/missy/materials/MZ/documents/frageboegen (accessed on 08.08.2024).
13. Rammstedt B.; Kemper C.; Klein M.; Beierlein C.; Kovaleva A. [A brief scale for measuring the five dimensions of personality: Big Five Inventory-10 (BFI-10)]. Eine kurze Skala zur Messung der fünf Dimensionen der Persönlichkeit: Big-Five-Inventory-10 (BFI-10). Köln: GESIS – Leibniz-Institut für Sozialwissenschaften; 2012.
14. Kemper C.; Beierlein C.; Bensch D.; Kovaleva A.; Rammstedt B. [A Short Scale for Measuring the Gamma Factor of Socially Desirable Response Behavior The Social Desirability Gamma Short Scale (KSE-G)]. Eine Kurzskala zur Erfassung des Gamma-Faktors sozial erwünschten Antwortverhaltens: Die Kurzskala Soziale Erwünschtheit-Gamma (KSE-G). Köln: GESIS – Leibniz-Institut für Sozialwissenschaften; 2012.
15. Council of the European Communities. Commission Recommendation of 6 May 2003 Concerning the Definition of Micro, Small and Medium-Sized Enterprises *Off J Eur Union* 2003, *L124,* 36–41.

16. DESTATIS. [Classification of economic activities, 2008 edition (WZ 2008)]. Klassifikation der Wirtschaftszweige, Ausgabe 2008 (WZ 2008). Available online: https://www.destatis.de/DE/Methoden/Klassifikationen/Gueter-Wirtschaftsklassifikationen/klassifikation-wz-2008.html (accessed on 08.08.2024).
17. German Social Accident Insurance (DGUV). Companies by size in 2023. Accident insurance in industrial sector. Available online: https://www.dguv.de/en/facts-figures/insured-person-companies/company-size/index.jsp (accessed on 02.10.2024).
18. Völter-Mahlknecht S.; Michaelis M.; Preiser C.; Blomberg N.; Rieger M. Forschungsbericht 448. Inanspruchnahme von Angebotsuntersuchungen in der arbeitsmedizinischen Vorsorge [Research Report 448. Use of optional examinations in occupational health prevention]: Bundesministerium für Arbeit und Soziales (BMAS); 2011.
19. Freiburger Forschungsstelle für Arbeitswissenschaften GmbH (FFAW). COPSOQ - Die Befragung zu psychischen Belastungen am Arbeitsplatz [COPSOQ - Questionnaire about work-related psychosocial demands]. Accessible online: https://www.copsoq.de/kontakt/ (accessed on 12.08.2024). 2020.
20. Beck D.; Lenhardt U. Consideration of psychosocial factors in workplace risk assessments: findings from a company survey in Germany. *International Archives of Occupational and Environmental Health* 2019, *92,* 435-451.
21. Ochsmann E. [Longitudinal Study onWork and Health under the Impact of the COVID-19 Pandemic]. Längsschnittstudie zu Arbeit und Gesundheit in Zeiten der Corona-Pandemie. Available online: https://www.uksh.de/arbeitsmedizin-luebeck/Forschung/Forschungsprojekte.html (accessed on 08.08.2024).
22. Sommer S.; Schmitt-Howe B. [Company and employee survey 2015 as part of the evaluation of the Joint German Occupational Safety and Health Strategy]. Betriebs- und Beschäftigtenbefragung 2015 im Rahmen der Dachevaluation der Gemeinsamen Deutschen Arbeitsschutzstrategie. Available online: https://search.gesis.org/research_data/ZA6759 (accessed on 08.08.2024).
23. Robert Koch-Institut (RKI). [Coronavirus SARS-CoV-2—Information and Guidance for People at Increased Risk of Severe Disease Progression COVID-19: Groups of People Known to be at Increased Risk of Severe Disease Progression 2020]. Coronavirus SARS-CoV-2—Informationen und Hilfestellungen für Personen mit einem höheren Risiko für einen schweren COVID-19- Krankheitsverlauf : Personengruppen, die nach bisherigen Erkenntnissen ein höheres Risiko für einen schweren Krankheitsverlauf haben. 2020. Available online: https://www.rki.de/DE/Content/InfAZ/N/Neuartiges_Coronavirus/Risikogruppen.html (accessed on 14.03.2021).
